# A Review of Machine Learning-Assisted Gas Sensor Arrays in Medical Diagnosis

**DOI:** 10.3390/bios15080548

**Published:** 2025-08-20

**Authors:** Yueting Yu, Xin Cao, Chenxi Li, Mingyue Zhou, Tianyu Liu, Jiang Liu, Lu Zhang

**Affiliations:** 1 School of Pharmaceutical Sciences and Institute of Materia Medica, Xinjiang University, Urumqi 830017, Chinacaoxin@stu.xju.edu.cn (X.C.); liutianyu@stu.xju.edu.cn (T.L.); 2 Key Laboratory of Xinjiang Phytomedicine Resources for Ministry of Education, School of Pharmacy, Shihezi University, Shihezi 832000, China

**Keywords:** machine learning, volatile organic compounds, non-invasive diagnosis, electronic

## Abstract

Volatile organic compounds (VOCs) present in human exhaled breath have emerged as promising biomarkers for non-invasive disease diagnosis. However, traditional VOC detection technology that relies on large instruments is not widely used due to high costs and cumbersome testing processes. Machine learning-assisted gas sensor arrays offer a compelling alternative by enabling the accurate identification of complex VOC mixtures through collaborative multi-sensor detection and advanced algorithmic analysis. This work systematically reviews the advanced applications of machine learning-assisted gas sensor arrays in medical diagnosis. The types and principles of sensors commonly employed for disease diagnosis are summarized, such as electrochemical, optical, and semiconductor sensors. Machine learning methods that can be used to improve the recognition ability of sensor arrays are systematically listed, including support vector machines (SVM), random forests (RF), artificial neural networks (ANN), and principal component analysis (PCA). In addition, the research progress of sensor arrays combined with specific algorithms in the diagnosis of respiratory, metabolism and nutrition, hepatobiliary, gastrointestinal, and nervous system diseases is also discussed. Finally, we highlight current challenges associated with machine learning-assisted gas sensors and propose feasible directions for future improvement.

## 1. Introduction

Disease diagnosis is the fundamental principle of clinical medicine. The application of systematic methods, including clinical symptom assessment, laboratory testing, and imaging analysis, helps to accurately determine the pathological mechanisms of the disease. This provides a scientific basis for subsequent treatment interventions. Early diagnosis is a critical component of this strategy, as it facilitates the effective enhancement of treatment outcomes and a reduction in medical expenditures. According to the World Health Organization (WHO), approximately 70% of patients with chronic diseases worldwide missed the optimal time for treatment due to the absence of effective early screening methods, leading to the progression of the disease to an irreversible stage. For instance, the five-year survival rate for early-stage lung cancer (e.g., stage IA) was over 90% after surgical resection. In contrast, the survival rate for stage IV patients was less than 10% [1,2]. Traditional medical tests include routine blood tests (red blood cell count, hemoglobin, white blood cell count, platelet count, etc.), routine urinalysis (urine red blood cell count, white blood cell count, urine protein, etc.), and imaging tests (X-rays, computed tomography (CT), magnetic resonance imaging (MRI), etc.), and endoscopy (gastroscopy, colonoscopy, bronchoscopy, laryngoscopy, etc.), and they are highly precise. However, they are invasiveness, time-consuming, and high-cost, which limits their use in early screening and real-time monitoring.

The concentration and type of volatile organic compounds (VOCs) in human exhaled breath are closely related to human health. Studies have shown that more than 3000 VOCs are produced during human metabolism, with concentrations ranging from parts per trillion (ppt) to parts per million (ppm) [3]. Changes in the types and concentrations of VOCs are significantly associated with over 50 diseases, including lung cancer, diabetes, and kidney dysfunction [4,5]. For example, acetone concentration in the breath of diabetics exceeded 1.8 ppm, which was much higher than the 300–900 ppb found in healthy individuals [6]. Traditional gas detection methods mainly include techniques such as gas chromatography mass spectrometry (GC-MS) [7,8,9], high performance liquid chromatography (HPLC) [10,11,12,13], infrared spectroscopy (IR) [14,15], chemical detector tubes, photoionization detector (PID) [16,17], and nuclear magnetic resonance (NMR) spectroscopy [18,19,20,21]. Each method has its own characteristics in terms of principle and application, but there are also significant limitations. GC-MS combines chromatographic separation and mass spectrometry to detect thousands of VOCs with ppb-level sensitivity. It also avoids cross-contamination and accurately identifies isomers [22]. However, it requires a high laboratory environment and relies on a pre-concentration step [23], making real-time monitoring impossible. The equipment is expensive, and a single test can take tens of minutes to hours, rendering it difficult to meet the demand for real-time clinical monitoring. HPLC is a highly advantageous analytical technique that has been widely recognized for its effectiveness in detecting highly polar, thermally unstable, or macromolecular compounds. It is characterized by high sensitivity and the capability to perform simultaneous analysis of multiple analytes, making it an indispensable tool across various scientific disciplines. However, certain limitations exist, including the time-intensive nature of the gradient elution process, high equipment costs, challenges in the detection of highly volatile compounds, and the potential occurrence of matrix effects [24]. IR utilizes the absorption properties of gas molecules at specific infrared wavelengths for detection and is suitable for the identification of the known compounds [25]. Different functional groups have characteristic absorption at specific wavelengths, while samples can be measured directly without changing chemical properties. However, highly volatile organic compounds (such as toluene and benzene) easily evaporate during sampling or pretreatment and are susceptible to interference from environmental humidity and dust [26]. This limits the accuracy of low-concentration gas detection. PID achieves a millisecond-level response through the principle of ultraviolet (UV) ionization. However, its response to gas mixtures is the weighted sum of all detectable compounds, so it cannot distinguish specific components. Furthermore, frequent calibration is required to maintain accuracy, which increases the cost of ownership of the equipment [16]. Although NMR can analyze various gases without damaging the samples and without pretreatment. However, its detection limit is relatively high, and it is usually not applicable for the effective analysis of trace substances with concentrations below 1 μM. It is less effective in analyzing compounds with low volatility or high molecular weight (e.g., certain lipids and steroids) [27]. In conclusion, traditional methods typically feature limited multicomponent analysis capabilities, highly specialized operations, poor real-time performance, and complex equipment. These shortcomings are particularly evident in trace and dynamic detection scenarios required for early disease screening. To achieve a breakthrough, there is a need for more time-efficient, miniature, and convenient detection technologies.

Gas sensors are core devices that convert information about gas composition and concentration into electrical signals. Their operating principle is based on the interaction between gas molecules and sensitive materials. Parameters used to evaluate sensor performance include sensitivity, selectivity, accuracy, limit of detection (LOD), resolution, reversibility, and response and recovery times [28]. Gas sensors have the advantage of being able to detect a single gas due to their small size and fast response time [29,30]. These sensors are widely used for combustible gas leakage detection [31], toxic and corrosive gas monitoring [32], exhaust gas detection [33], VOCs detection, air quality sensing [34], and breath analysis [35]. In recent years especially, using gas sensors to diagnose different types of diseases has become a popular area of research. However, single sensors have significant drawbacks. They typically have only a selective response to a single gas and are susceptible to interference from non-target gases. In addition, it is difficult to detect a series of volatile organic compounds associated with disease [36]. Since only the concentration data of a single gas can be provided, a multidimensional feature space related to the disease cannot be constructed. Consequently, the risk of underdiagnosis is high. For instance, the concentration of trans-2-pentene, an early lung cancer marker, is just 0.30 ppb [37]. However, a noisy signal from a single sensor often obscures effective information, resulting in a higher rate of missed diagnoses. A sensor array is a device composed of multiple sensors that can detect various types of gases. Compared to a single gas sensor, it provides as much chemical diversity as possible [38]. The sensors in an array typically respond to a unique set of signals called a “gas fingerprint” [39]. The array utilizes the cross-sensitivity [40] properties of the sensors to construct multidimensional response signals, accurately resolving mixed gas compositions that traditional single sensors cannot distinguish. Although gas sensing arrays can expand the detection dimension through multi-source sensing, the bottleneck lies in the inherent cross-sensitivity of the sensors with baseline drift [41] under environmental perturbations, inter-sensor signal coupling, and noise interference [42]. High-dimensional nonlinear responses are difficult to be efficiently solved by traditional chemometrics.

**Figure 1:**
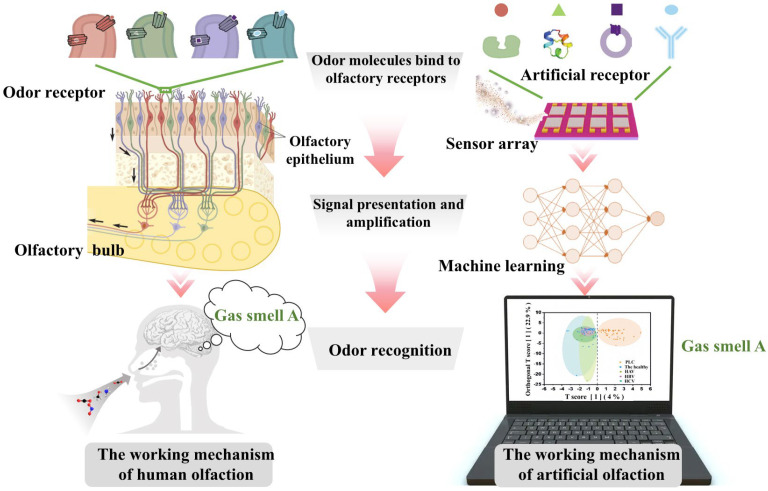
Comparison chart of the working modes of human olfaction and electronic nose.

Machine learning algorithms can convert sensor signals into distinguishable patterns using deep feature extraction and pattern recognition. They can even extract features from time series and raw sensor data to identify various VOCs in mixed components. Machine learning can be broadly categorized as either supervised or unsupervised. Supervised learning involves mapping input data to output based on training data. In unsupervised learning, machine learning systems are provided with unlabeled datasets, so they have no predetermined results and only extract information and patterns from raw data through algorithms [43]. Gas sensor arrays resemble olfactory receptor neurons (ORNs) in their properties. Each ORN expresses one genetically encoded receptor, so its selectivity is limited [44]. ORNs as a whole can recognize specific compounds or mixtures of compounds. Similar to the way ORNs work, gas sensing arrays combined with the appropriate machine learning algorithms have been called “electronic noses” [45]. The human olfactory system and the electronic nose have a core bionic correspondence in their mechanisms of operation, but there are also essential differences. Both follow the basic process of “detection–signal processing–recognition,” aiming to convert complex odor information into recognizable signals. The key difference lies in the “core processor” that handles the signals. The human body relies on highly complex biological neural networks and the brain’s cognitive power, while the electronic nose relies on artificially designed electronic circuits and machine learning models for computation and pattern recognition. (The comparison is shown in Figure 1). In other words, the e-nose is a biomimetic system inspired by the biological sense of smell and realized through engineering. The “receptors” are artificial, and electronics perform the functions of the “olfactory bulb” and “brain”. Electronic signal processing and algorithms replace the functions of the olfactory bulb and brain. Electronic noses are effective tools for classifying different VOCs. They can adequately distinguish between samples and situations involving many different compounds. An electronic nose consists of the following three stages: gas detection by a sensor array, data processing, feature extraction, and pattern recognition [46,47]. Studies have shown that this e-nose system can achieve over 90% accuracy in discriminating between lung cancer and healthy breath samples [48], approaching the performance of conventional mass spectrometry. The Lind research team [49] has constructed a microhotplate array containing four sensors. They used chemical vapor deposition (CVD)-grown monolayer graphene as the base material. They functionalized three different types of metal oxide nanocoatings, CuO-MnO_2_, In_2_O_3_, and Sc_2_O_3_. This functionalization was carried out by pulsed laser deposition (PLD) technology. They have utilized the array to collect about 100 sets of gas cycle samples (30/60 ppb NO_2_, 30/60 ppb O_3_ and their gas mixtures) over a 72-h period, extract features such as response amplitude, response rate, and recovery rate. They employed a single hidden layer ANN as a machine learning model. The results showed that after adding the recovery rate features, the sensing array can classify the five gas environments with an accuracy of 94%.

**Figure 2:**
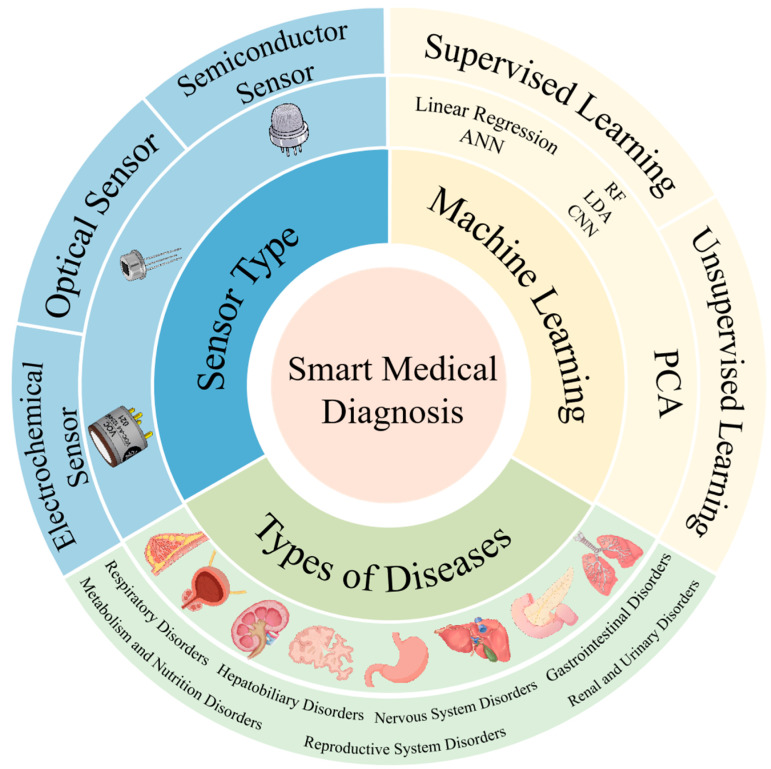
Machine learning-assisted gas sensor arrays for medical diagnosis.

Numerous reviews have classified gas sensors based on sensor type or gas markers. The review written by Pradyumn et al. [43] have explored the research progress of selective gas sensors. The paper also discussed how machine learning techniques can address cross-sensitivity issues in mixed gas environments by analyzing sensor response signals, enabling the precise classification of gas types and concentration prediction. Zong et al. [50] have systematically discussed the latest developments in smart gas sensors, focusing on their working principles based on advanced materials, structural design, and diverse applications in fields such as environmental monitoring, medical diagnosis, food safety, and public safety. At the same time, it provided an in-depth analysis of how sensor arrays can be combined with artificial intelligence algorithms and integrated with Internet of Things (IoT) technology. Different from them, this study focuses on the latest applications of integrating machine learning algorithms with gas sensor arrays in the field of medical diagnosis. The principles underlying the construction of electrochemical, optical, and semiconductor sensors for disease diagnosis are systematically outlined (Figure 2). The distinction between supervised and unsupervised learning is emphasized, and the role of machine learning in medical diagnosis is briefly described. The paper conducts an in-depth analysis of core algorithms for feature extraction and pattern recognition, such as linear models, decision trees, and neural networks. Unlike traditional literature that discusses individual technologies in isolation, this work emphasizes the synergistic mechanisms between sensor arrays and algorithms. By analyzing typical clinical cases, such as diabetes breath detection and lung cancer metabolomics analysis, the potential applications of intelligent sensing systems in multidimensional disease diagnosis are fully revealed.

## 2. Common Types of Gas Sensors

A gas sensor is a conversion tool that transforms parameters, such as gas volume fraction and concentration, into corresponding electrical signals [51]. This process offers advantages such as high sensitivity and a short response time. The main types of gas sensors used in clinical disease diagnosis are electrochemical, optical, and semiconductor sensors. This section introduces the principles, advantages, disadvantages, and application scenarios of these sensors.

### 2.1. Electrochemical Sensor

Electrochemical sensors are currently one of the most widely used types of sensors. Thanks to their excellent selectivity, high sensitivity, and low-cost detection capabilities, these devices are used in a wide range of applications across fields such as biomedicine, environmental science, and materials science [52]. An electrochemical gas sensor is a small electronic device based on electrochemical principles that is used to detect the concentration of specific gases. Electrochemical sensors measure the concentration of a substance by detecting the current signal generated when target gas molecules undergo a redox reaction on the surface of the working electrode. A working electrode, a counter electrode, and a reference electrode are the usual components, and they are filled with a specific electrolyte [53]. Electrochemical gas sensors, as exemplified by breath nitric oxide (NO) content detection, have become an important tool in asthma management. In the study by Horváth et al., the detection limit can reach the ppb level (0.1–300 ppb) [54]. Electrochemical sensors show excellent response to electrochemically active gas molecules such as NO and ethanol and are widely used in medical applications such as breath alcohol detection and acetone monitoring for diabetic ketoacidosis. In addition, integrating electrochemical biosensors into portable, implantable, and wearable devices enables convenient multi-parameter detection.

**Figure 3:**
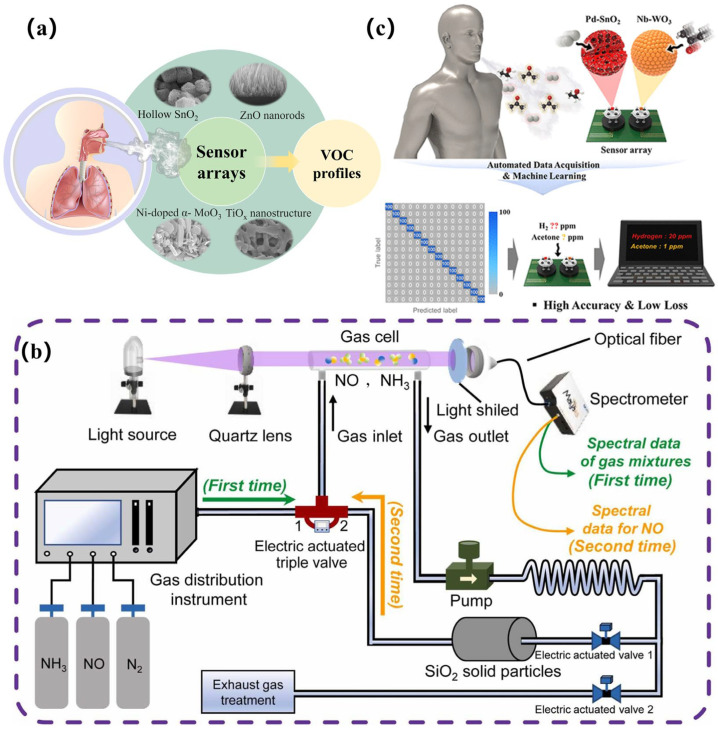
(**a**) Array of gas sensors with different catalytic materials. (**b**) Schematic diagram of breath analysis based on semiconductor sensor arrays and machine learning [62]. (**c**) Schematic diagram of the new type of chemical resistance detector [90].

Currently, research on electrochemical sensors is focused on improving multi-parameter joint detection capabilities by constructing sensor arrays containing different catalytic materials (Figure 3a). Combined with pattern recognition algorithms, multiple biomarkers in exhaled breath can be analyzed simultaneously [55]. With this technological breakthrough, the inflammatory marker NO and volatile organic compounds produced by metabolic abnormalities (like acetone and isoprene) can be detected by a single sensor module [56], providing more comprehensive data support for the differential diagnosis of diseases.

### 2.2. Optical Sensor

Optical sensors measure concentration by detecting the interaction between gas molecules and light waves. The fact that different gases selectively absorb or scatter light of specific wavelengths is what this principle is based on. The sensors in question generally comprise a light source, a gas chamber, an optical filter, and a photodetector. When the target gas enters the detection chamber, it causes a change in light intensity, wavelength, or phase, creating an analyzable signal. Machine learning algorithms can analyze these signals to achieve high-precision monitoring of gas concentration. For example, Wang et al. [57] have constructed an infrared sensor system for detecting various gases, including CH_4_, CO, and CO_2_. The researchers designed a direct laser absorption spectroscopy structure based on interbank cascade lasers and used a recursive least-square adaptive denoising algorithm as a way to monitor CH_4_. Consequently, they were able to significantly enhance the test’s sensitivity, achieving a detection limit of 43.9 ppb within 6 s and 6.3 ppb within 240 s. The highly accurate monitoring of two different CO gas concentrations, with determination results of 512.2 ± 3.04 ppm and 2513.5 ± 2.4 ppm. There are many types of optical sensors, including infrared and laser spectral sensors, fluorescent sensors, and fiber optic sensors [58]. In the field of medical diagnostics, IR sensors are among the most commonly used. They utilize the characteristic absorption peaks of gas molecules in the mid-infrared band for identification. For example, the strong absorption band of carbon dioxide at 4.26 μm is a widely used property in the development of respiratory function monitoring devices. Fluorescence sensors mainly rely on exciting specific gas molecules with light. When UV light irradiates the gas under test, molecules with fluorescent properties, such as NO, emit fluorescence at specific wavelengths. The fluorescence intensity value corresponds to the concentration of the gas [59]. This type of detection uniquely advantages the monitoring of airway inflammation in asthmatics and is not affected by other common components of exhaled breath. Fiber optic sensors [60] have developed rapidly in recent years. These sensors integrate sensing materials onto the surface of optical fibers to form functionalized probes. When a target gas molecule is adsorbed onto the surface of a fiber coated with a sensitive material, it alters the light conduction characteristics. This structure is ideal for creating miniature detection probes that can be inserted directly into breathing masks for real-time monitoring. The detection limit of the lung cancer marker pentane is reduced by modifying the metal–organic framework material on the surface of the optical fiber [61], meeting the sensitivity requirements for early screening. Similarly, an optical chemical gas sensor system based on spectral autocorrelation has been proposed by Zhu et al. [62] in Figure 3b, which combines a convolutional neural network (CNN) algorithm. It has achieved stable and reliable detection of NO and NH_3_ in breath, with a detection limit of up to ppb level.

As optoelectronic integration technology and artificial intelligence algorithms progress, optical sensing technology is developing toward miniaturization and intelligence. However, it still faces technical challenges, such as environmental interference and cross-sensitivity. In the future, technologies such as lab-on-a-chip, artificial intelligence, and quantum sensing will be broken through. Consequently, optical gas sensors are poised to assume an increasingly pivotal role in the realm of early disease diagnosis and health monitoring.

### 2.3. Semiconductor Sensor

Semiconductor sensors are devices that detect gas molecules through the interaction of a sensitive element with molecules that either “passively diffuse” or “actively inhale” into the sensing area. Changes in electrical properties, including resistance, capacitance, and field effects, are triggered by this interaction. Concurrently, information about the type and concentration of the gas is converted into electrical signals. The gas composition and concentration can then be ascertained through the conversion of the information into electrical signals. Semiconductor gas sensors can be classified by material type, into metal oxide semiconductor (MOS) sensors [63], organic semiconductor sensors [64,65], carbon matrix composite sensors [66,67,68], and nanomaterial sensors [66,69]. These sensors offer high sensitivity, a fast response time, and high accuracy. They are also recyclable and low-cost. The core of their technology lies in the realization of trace gas detection through changes in surface conductivity triggered by gas adsorption.

Conventional resistive sensors are divided into n-type and p-type sensitive element materials. N-type semiconductors are often made of metal oxides, such as SnO_2_ [70], ZnO [71], tungsten trioxide (WO_3_) [72], and TiO_2_ [73], or metal sulfides, such as SnS_2_ [74], MoS_2_ [75], and InSe [76,77]. Their electrical conductivity is dominated by free electrons inside the material. P-type semiconductor gas sensors achieve gas detection through hole concentration modulation. Metal oxides, including but not limited to CuO [78], NiO [79], Co_3_O_4_ [80], and Cr_2_O_3_ [81], and organics, such as organometallic frameworks and polypyrenes, are typical representatives of p-type semiconductors. Their electrical conductivity is dominated by holes inside the material. N-type materials form hybrid heterojunctions with p-type materials. These hybrid heterojunctions can be pn junction or isotype heterojunction types. The pn junction semiconductors mainly work based on interfacial energy band modulation and the synergistic charge transfer effect [82]. Because of their different Fermi energy levels, when the two semiconductors are in contact, electrons flow from the n-type semiconductor to the p-type semiconductor, while holes migrate in the opposite direction. This forms a built-in electric field and an interfacial depletion layer. Isotype heterojunctions [83] usually refer to interfaces formed from the same semiconductor material type (e.g., n-n or p-p) with different doping concentrations, crystalline phases, or nanostructures. Isotype heterojunctions exhibit similar charge transfer and interface depletion layer mechanisms. However, their same type of conductivity results in a smaller interfacial energy band shift [84]. The formation of narrow depletion layers with unilateral distributions is observed when the difference in Fermi energy levels is smaller than the n-p type. Thus, the barrier height for carriers crossing the interface decreases significantly, enabling the sensor to efficiently separate charges at low driving voltages [85]. Additionally, the selectivity and responsiveness of the material system can be substantially enhanced by noble metal modifications (e.g., platinum [86], gold [87,88]). For example, Righetton et al. [89] have developed a chemoresistive sensor based on pure WO_3_ and Si-hybridized WO_3_ nanoparticles, which can be used for non-invasive diabetes detection. This semiconductor sensor exhibits high selectivity and sensitivity, with acetone detection as low as 20 ppb. When combined with algorithms, semiconductor sensors will achieve further breakthroughs, effectively improving the accuracy of disease differentiation. Nam et al. [90] have constructed a semiconductor metal oxide sensor array for efficient disease prediction, as shown in Figure 3c. In this study, SnO_2_ and WO_3_ sensors were combined with algorithms such as PCA and CNN to achieve high-accuracy detection. At 325 °C, a combination of 10% Pd-SnO_2_ and 10% Nb-WO_3_, when combined with a 1D CNN, achieved 100% accuracy. Additionally, the loss in this process was as low as 1.6 × 10^−3^, enabling error-free prediction.

Currently, these sensors are characterized by poor selectivity and susceptibility to interference from cross-sensitive gases. MOS sensors require continuous heating over a long period of time to maintain activity [63], resulting in higher power consumption and increased susceptibility to aging damage. Semiconductor gas sensors have outstanding advantages in terms of portability and real-time monitoring. However, their reliability and long-term stability in complex environments still need to be optimized.

## 3. Machine Learning for Gas Sensors

Expert systems, machine learning, evolutionary computing, fuzzy logic, computer vision, natural language processing, and recommendation systems are some of the main fields of artificial intelligence study. As one of the fastest-growing technological domains of our time, machine learning [91,92,93] is central to artificial intelligence and data science [94] and sits at the nexus of computer science and statistics. The four main categories of machine learning are reinforcement learning, semi-supervised learning, unsupervised learning, and supervised learning. Currently, in the medical field, the primary applications of machine learning in gas sensor detection are supervised learning and unsupervised learning. The signals collected by gas sensors are usually characterized by high dimensionality, nonlinearity, and high noise interference, while machine learning can process the signals to form a synergistic mechanism. On the one hand, machine learning improves the monitoring performance of the sensing system, such as accuracy. On the other hand, it promotes the intelligent, modernized, portable, and accurate development of medical gas sensing.

### 3.1. Supervised Learning

Supervised learning [95,96] is a technique that uses labeled data to train models so that they can make predictions on new data. The main steps include data collection, data preprocessing, model selection, model training, model evaluation, parameter adjustment, and model deployment. In the supervised learning process, one or more algorithms are typically selected to better be used to develop the prediction model. Supervised learning methodologies encompass linear regression, SVM, ANN, and RF algorithms.

#### 3.1.1. Linear Regression

**Figure 4:**
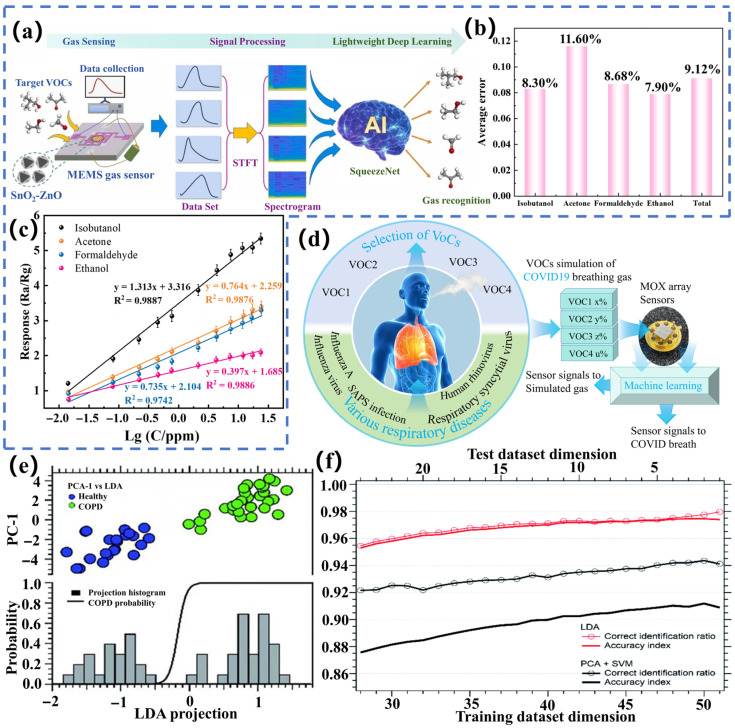
(**a**) Schematic representation of the VOC compound detection system utilizing micro-electro-mechanical system gas sensors [100]. (**b**) Average error in concentration prediction. (**c**) Fitting equations for four VOCs. (**d**) Schematic representation of the detection of COVID-19 patients. (**e**) The first PCA component and LDA prediction. (**f**) Recognition rate (dashed line) and accuracy index (solid line) of PCA-SVM (black) and LDA (red) under different datasets [115].

Linear regression [97] is a fundamental supervised learning technique rooted in statistical learning, which defines the connection between input variables and target variables by formulating a linear equation. As a commonly adopted statistical method, it has demonstrated significant efficacy across diverse applications [98]. Zhao et al. [99] have proposed a signal processing approach based on stochastic contact regularized linear regression, which was applied to investigate signal inconsistencies in gas sensors. This methodology was validated through experiments conducted with various sensors, including WO_3_, ZnO, Fe_2_O_3_, and SnO_2_. Figure 4a illustrates an e-nose system based on MEMS gas sensors constructed by Tang et al. [100], which used linear regression algorithms for VOCs concentration prediction. The response curves in the dataset revealed a significant linear correlation between the logarithm of concentration and the responses elicited by four substances, isobutanol, acetone, formaldehyde, and ethanol. Figure 4b shows the fitted equation demonstrating gas detection at the ppm level. Under linear regression, the *R*^2^ values for the four-target volatile organic compounds are 0.9799, 0.9768, 0.9888, and 0.9515, respectively, with corresponding average prediction errors of 0.1208, 0.1169, 0.1058, and 0.1363. Figure 4c demonstrates the effectiveness of this linear regression model in integrating VOCs.

#### 3.1.2. Support Vector Machine

SVM is a powerful supervised learning algorithm that is broadly categorized into two types, support vector regression (SVR) [101,102,103] and support vector classification (SVC). SVR, an extension of SVM for regression tasks, is applicable to a wide range of problems. Conversely, support vector classification is specifically designed for classification tasks, with a primary focus on addressing binary classification problems. In the context of practical applications, Liu et al. [104] have employed an SVM algorithm to process signals from an electronic nose for a wine classification experiment. Features derived from the electronic nose signals served as input vectors for the SVM, which was then trained to distinguish between various types of wine. During training, known-category alcohol samples were used to train the SVM, enabling it to learn the feature patterns of different alcohol signals and thereby achieve accurate classification of unknown wine samples. SVM and LVQ have been used by Harakeh et al. [105] to identify single gases such as Ar, O_2_, He, and CO_2_, achieving 100% learning and validation rates. Extremely high accuracy and reliability were demonstrated by the SVM algorithm in precisely classifying single gases. This performance lays a solid foundation for further research and optimization of gas identification algorithms. Meanwhile, it provides valuable reference for the development of algorithms dedicated to identifying multiple mixed gases. Support vector machines have widespread applications in gas detection. Han et al. [106] have constructed a sensor array for detecting mixed gases and used an algorithm based on kernel principal component analysis and SVC for identifying the components of mixed gases. In this process, the model learned from labeled data to achieve identification of different components in mixed gases.

#### 3.1.3. Artificial Neural Network

ANN is a deep learning architecture that mimics biological neurons and can learn complex nonlinear mappings between input and output by adjusting connection weights. For example, data features are automatically learned by ANN algorithms through multi-layer nonlinear structures, demonstrating strong pattern recognition capabilities. Zampolli et al. [107] have achieved efficient classification of high-dimensional sensor data using ANNs. Additionally, by combining temperature cycling and impedance spectroscopy techniques, the accuracy of gas identification has been significantly improved by the ANN model. Habib et al. [108] have used a dataset from a network of six gas sensors and applied neural networks for gas identification, achieving an accuracy rate of 85.3%. Khan et al. [109] have processed sensor array data based on GaN nanowires using multiple classification algorithms to detect cross-sensitive gases. Among these, the ANN algorithm played a crucial role in identifying target gases. Figure 4d shows that Zou et al. [110] have constructed a machine learning-based sensing system, with the ANN regression algorithm predicting the concentrations of nine VOCs. After 10,000 training iterations, the system demonstrated excellent training performance, with the training curve converging to approximately 100% prediction accuracy and the validation curve converging to approximately 0% loss function. Compared to the cross-validation *R*^2^ value of 0.76 for the commercial sensor array for nine VOCs, the AIST sensor achieved an *R*^2^ value of 0.97, demonstrating superior predictive performance.

#### 3.1.4. Random Forest

RF [111] is recognized as a representative conventional machine learning algorithm that improves model performance by building multiple decision trees and combining their predictive outcomes. The randomization process utilizes the variability among decision trees, thereby improving the overall performance of the RF model. For example, Wiederoder et al. [112] have used an array of 12 sensors based on an IDE electrode structure, with graphene nanoplate polymers as the sensing material. Measurement data for different analytes were reduced in dimension using PCA, and four different machine learning algorithms, including RF, were employed for analysis. During the experiment, RF demonstrated a high classification accuracy rate. Christinelli et al. [113] have utilized models such as MLP and RF for multi-objective regression in their study using an electronic tongue to detect endocrine disruptors. They also predicted the concentrations of individual endocrine disruptors and their mixtures, evaluating model performance by comparing root mean square errors. In the study by Zou et al. [110], the RF algorithm was used to classify nine VOCs, and feature importance analysis was obtained. Throughout this process, five-fold cross-validation was utilized to evaluate the model, and recursive feature elimination cross-validation was implemented to determine the classification accuracy of the sensor array. The results indicated that classification accuracy rates of 96% and 100% were achieved by commercial sensors and AIST sensors, respectively, with superior classification performance being demonstrated by the AIST sensors.

#### 3.1.5. Linear Discriminant Analysis

Linear discriminant analysis (LDA) is a powerful statistical method [114] used to extract redundant and noisy information from raw datasets into their core features. It is worth noting that LDA belongs to supervised learning and has been widely applied in data dimensionality reduction to reveal potential structures and patterns in data. LDA is also frequently applied in gas sensor data analysis processes and has achieved high recognition accuracy. For example, Drera et al. [115] have used the LDA algorithm to analyze 52 exhaled gas samples, demonstrating the algorithm’s exceptional performance. Compared to the PCA algorithm used in the experiment, LDA achieved better clustering effects by defining directions in the feature space and exhibited high recognition accuracy for the data as shown in Figure 4e. In the case of a reduced dataset, the recognition ratio of the samples by LDA is greater than 95%, and the accuracy is highly similar to the recognition ratio, with an overall accuracy of 99%. Figure 4f demonstrates that the accuracy and stability of LDA is significantly higher than that of PCA + SVM in the classification process of chronic obstructive pulmonary disease (COPD) samples. In the study by Reena Thriumani et al. [116], data analysis was conducted on VOCs released by lung cancer cell lines (MCF7) and control cell lines (WI38VA13) detected by gas sensors. In this study, LDA analysis played a central supporting role, primarily in feature dimension reduction, classification model construction, and result validation. The decision function for MCF7, WI38VA13, and other samples achieved 100% accuracy, with clear clustering of different cell line samples and good separation performance. In the four classifier tests based on LDA, the accuracy, sensitivity, specificity, and precision of the SVM, PNN, and KNN classifiers all reached 90%, demonstrating high predictive quality.

### 3.2. Unsupervised Learning

Unsupervised learning [117,118] involves training using unlabeled data, where the dataset contains only input features. Without explicit output labels or target values, the model’s task is to discover structures and patterns within the data. PCA is one of the most common unsupervised dimension reduction algorithms and has been applied in gas sensors. This method facilitates the projection of high-dimensional data onto a lower-dimensional subspace via orthogonal transformation, thereby retaining the maximal variance inherent in the original dataset. Without explicit output labels or target values, the model’s task is to discover structures and patterns in the data. However, PCA is unable to capture nonlinear relationships and has poor interference immunity, so it needs to be used in combination with LDA and SVM to make up for its shortcomings. For example, in a VOC identification experiment based on multiple overlapping sniffing strategy, Liu et al. [104] have used PCA to process the data. In addition, PCA was used to reduce the dimensionality of the feature, demonstrating the ability to identify different VOCs. Lee et al. [119] have normalized sensor response data to reduce data variance. Similarly, Danieli et al. [120] have employed PCA to simplify data analysis, reduce computational costs, and remove noise and redundant information when processing gas sensor data. PCA was employed to reduce the dimensionality of sensor signal features, transforming the original feature space into the principal component space. This approach reduces data dimensions while retaining most of the data information, facilitating subsequent regression analysis. In the study by Mahdavi et al. [121], PCA has been used for comparison with other feature selection methods. It performed slightly worse than the proposed SVM and KNN feature selection methods in terms of accuracy, sensitivity, and specificity. However, the PCA algorithm also demonstrated the role of unsupervised learning in handling high-dimensional data, such as simplifying classification models and reducing training time. High-dimensional data generated by gas sensor arrays often contains errors induced by external environmental interference. PCA can summarize the correlations between sensors and calculate the principal component directions. It can also distinguish gas categories in a two-dimensional space and preprocess data as input for other algorithms such as SVM and RF. Chang et al. [122] have constructed a gas analysis system comprising seven metal oxide sensors. Combining PCA dimensionality reduction with LDA classification algorithms, pattern recognition was performed on VOCs from 37 non-small cell lung cancer patients (81.1% at stages I–II) and 48 healthy volunteers. T ng cancer patients significantly shifted toward the healthy control group. This system can achieve early diagnosis of lung cancer and postoperative prognosis assessment through the dynamic monitoring of VOC change. Zou et al. [110] have used PCA algorithms to analyze datasets obtained from commercial sensors and AIST-made sensor arrays, exploring and comparing the characteristics of the two types of sensors. PCA results showed that AIST sensors had a distinct advantage in distinguishing between nine types of VOCs. The PCA scores of the commercial sensor array for some VOCs were clustered and overlapped in three quadrants, posing challenges in differentiation. In contrast, the PCA scores of the AIST sensor array did not overlap, enabling better classification of the nine VOCs. Similarly, in the study by Giovanni Drera et al. [115], the PCA algorithm has been applied to analyze 26 test datasets for NH_3_, NO_2_, H_2_S, and C_6_H_6_ gases. NH_3_ and NO_2_ can be effectively separated by the sensors, and the four gases exhibit good trends in the PCA space.

## 4. Application in Disease Diagnosis

The transition from single-marker detection to multidimensional illness diagnosis has been made possible by the combination of gas sensor arrays and machine learning techniques. The therapeutic uses of this technology for disorders of the respiratory, metabolism and nutrition, hepatobiliary, gastrointestinal, nervous system, and renal, urinary, and reproductive system disorders are methodically covered in this chapter. The chapter proves the crucial importance of machine learning models in feature categorization and shows how sensor arrays can capture disease-specific VOC fingerprints by profiling the properties of biomarkers in typical situations (Table 1).

### 4.1. Respiratory Disorder

As the primary mechanism for gas exchange between the human body and the external environment, the respiratory system is frequently linked to severe illnesses such asthma, COPD, and lung cancer. These disorders have a close relationship with abnormalities in respiratory function. These illnesses have subtle early signs that are easy to ignore. Conventional diagnostic techniques, like imaging and pulmonary function testing, have drawbacks, such as being intrusive and lacking sensitivity. Many patients miss the best period for therapy as a result of these restrictions.

#### 4.1.1. Lung Cancer and Chronic Obstructive Pulmonary Disease

Lung cancer has the highest mortality rate of all cancers worldwide. However, the 7th edition of the TNM staging system [148] shows that patients in the early stages (stages I–IIA) have higher survival and cure rates with adequate treatment. Early diagnosis and treatment are effective ways to intervene in lung disease [149]. Early symptoms are nonspecific, including persistent cough (especially with bloody sputum), chest pain, and dyspnea [150,151]. The lungs lack pain-sensing nerves, allowing tumors to grow insidiously until the advanced stage. Emphysema, persistent bronchitis, and airflow restriction are the hallmarks of COPD, a prevalent chronic illness today. It may progress to respiratory failure and pulmonary heart disease. The primary means of diagnosis are symptom evaluation and pulmonary function tests (such as the FEV1/FVC ratio) [152,153,154]. However, due to their lack of sensitivity, these techniques make it challenging to identify early disease progression. It is easy to mistake symptoms for asthma, which can result in misdiagnosis and other issues. Gas sensors for breath analysis can uncover indicators of COPD and lung cancer by real-time detection of VOCs in exhaled gas. The issue of individual sensors’ inadequate selectivity is effectively resolved by combining sensor arrays with pattern recognition algorithms.

**Figure 5:**
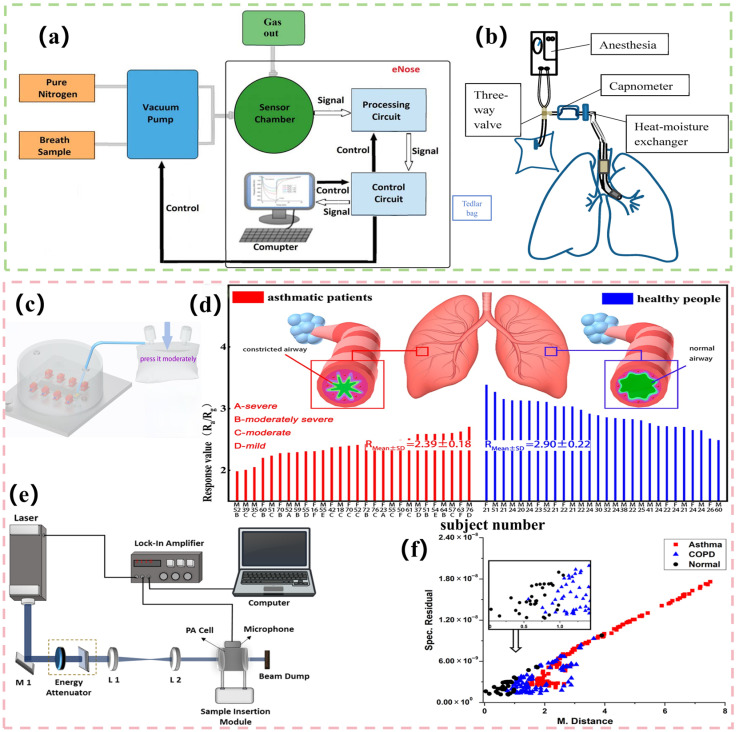
(**a**) The hardware composition of the electronic nose system, including the sensor array, processing circuit, control circuit, air pump, and computer. (**b**) System architecture diagram and sample collection [124]. (**c**) Schematic diagram of breath detection by sensor. (**d**) Comparison of the response values of exhaled breath between asthma patients and healthy individuals, marking gender and age, where M and F represent male and female, respectively. According to the BDT results, the severity is successively classified as A, B, C, D, E, and F, where E and F represent small airway ventilation dysfunction and normal lung ventilation but diagnosed with asthma, respectively [126]. (**e**) PAS sensor experimental setup. (**f**) Based on the “match/mismatch” test results of the normal calibration set, a display the distribution of the Mahalanobis distance and spectral residuals [127].

Highly sensitive sensor arrays are capable of capturing VOCs in real time from patients’ exhaled gases. Common biomarkers exhaled by lung cancer patients include styrene (normal values of 3.7–20 ppb [155]), alcohols (ethanol, isopropanol [156,157]), pentanes, aldehydes (glutaraldehyde, hexanaldehyde, nonanaldehyde), ketones (3-hydroxy-2-butanone), etc. [158], while COPD patients exhale isoprene, NO [159], benzaldehyde, o-xylene, and potential markers ethanol and methanol [160,161,162]. Among them, aldehydes exhaled by lung cancer patients are associated with lipid peroxidation, and ketones are associated with abnormal glucose metabolism, which may reflect the metabolic changes in tumor cells and are found in elevated concentrations in the exhaled breath of lung cancer patients; isoprene and benzaldehyde exhaled by COPD patients are associated with oxidative stress and inflammation. The sensor array responds selectively to the relevant markers. It converts chemical signals into electrical or optical signals. In addition, it generates multidimensional response features. The deep learning model is then used for processing such as feature extraction and pattern recognition, and a disease-specific classification model is constructed by comparing the distribution and dynamic pattern of VOC concentration in healthy populations and patients. The grading warning mechanism is triggered when the concentration of markers in VOCs is detected to be consistently above or below the threshold normal limit for lung cancer and COPD. Binson’s team [123] has designed an electronic nose system containing five MOS gas sensors (TGS 2600, TGS 2620, TGS 2610, TGS 822, and TGS 826), one hot-wire-type MR516 sensor, one ME4-C6H6 electrochemical sensor, and one NAP-55A catalytic sensor. All of the system’s hardware components and individual sensor response voltage variations are shown in Figure 5a. A total of 199 researchers were recruited for testing. Of these, 51 were lung cancer patients, 55 were COPD patients, and 93 were healthy controls (HC). Of the participants, 20% were set aside for the validation set, while the remaining 80% were chosen for the training set. This work included three integrated learning techniques, XGBoost, the adaptive boosting (AdaBoost) algorithm, and RF, in addition to the Kernel principal component analysis (KPCA) dimensionality reduction methodology for feature extraction. With 79.31% accuracy, 70.00% sensitivity, and 84.21% specificity for lung cancer diagnosis and 76.67% accuracy, 66.67% sensitivity, and 83.33% specificity for COPD detection, the XGBoost algorithm yielded the best classification results. The identification of lung cancer had an area under the curve (AUC) of 0.84, while the detection of COPD had an AUC of 0.76. Huang et al. [124] have used 32 highly sensitive, carbon nanotube-based conducting polymer (CP) sensors to create the Cyranose 320 electronic nose system, as shown in Figure 5b. Carbon nanotubes and polymers, such as poly-vinyl butyral, polyvinyl acetate, polystyrene, and polyethylene oxide, are the main components of this system. To create the prediction model, the study team integrated the LDA and SVM algorithms. For internal validation and training, they used 203 samples, comprising 147 controls and 56 lung cancer patients that were gathered between 2016 and 2017. In 2018, they recruited 41 samples (12 lung cancer patients and 29 control people) for external validation. The findings demonstrated that both the LDA and SVM internal and external validation AUC values were higher than 0.9. The sensitivity and specificity for LDA and SVM in the external validation were 75.0% and 96.6%, respectively, and 83.3% and 86.2%. This study passed rigorous cross-validation and the standardized sampling procedure. Yusuke’s team [125] has constructed a chemosensor array containing 12 channels using a membrane-based surface stress sensor (MSS) based on nanomechanical technology. Each channel was coated with different receptor materials, including silica/titanium dioxide mixed nanoparticles (STNPs) functionalized materials (such as octyl and phenyl modified STNPs) and a variety of commercially available polymers [163] (e.g., poly(ethylene oxide), polystyrene, poly(ethylene vinylidene fluoride), and poly(caprolactone)). They created chemically varied cross-response arrays by inkjet printing these compounds onto the sensor surface. In order to create a machine learning model, the researchers integrated RF classifiers, refined hyperparameters using five-fold cross-validation, and retrieved features using a combination of 12-channel signal differences. The preoperative and postoperative patients of 57 patients who underwent lung cancer surgery were included in the sample set as breath samples. The temperature and humidity were strictly controlled, and a repetitive measurement process was adopted to reduce interference. According to the results, a model with five channels (1, 4, 5, 6, and 7) produced positive and negative predictive values of 80.6% and 81.2%, respectively, in 100 validation repetitions, along with 80.9% accuracy, 83.0% sensitivity, and 80.7% specificity. The presence of residual cancer cells following surgery, which reduces prediction accuracy, a small sample size, a single research area, and an inadequate number of early-stage lung cancer patients are some of the drawbacks that these studies typically have. More research is required to determine whether they are globally generalizable. To increase the dependability of clinical applications in the future, multimodal validation, feature extraction optimization, and sample size expansion will all be required.

#### 4.1.2. Asthma

Asthma is a common chronic respiratory disease characterized by chronic inflammation of the airways, which causes them to become sensitive and swollen. The disease is characterized by recurrent episodes of wheezing, shortness of breath, chest tightness, and coughing, often occurring at night and in the early morning. Since asthma cannot be cured, early diagnosis and monitoring are essential to controlling the progression of symptoms. Traditional pulmonary function tests rely on patient cooperation, and bronchial provocation tests carry the risk of inducing acute attacks [164]. However, the development of sensor technology has provided a new avenue for noninvasive asthma testing, greatly improving the convenience and real-time nature of the process.

Breath testing sensors play an important role in asthma diagnosis. Exhaled breath nitric oxide (FeNO), H_2_S, and other endogenous VOCs that are not inhaled from the environment are key biomarkers reflecting airway inflammation [165]. Identified VOCs in asthmatics include branched hydrocarbons, methylated alkanes (e.g., 2,4-dimethylheptane, 2,3,5-trimethylheptane, and 3,7-dimethylnonane; octanal; nonanal; 2-octanal; 2-hexanone; 6,10-dimethyl-5,9-undecadien-2-one; butyric acid; isoprenebutyric acid; and isoprene [166,167,168,169]. FeNO is mainly produced by airway epithelial cells and inflammatory cells (e.g., neutrophils and eosinophils), driven by interleukin-4 (IL-4) and IL-13 [170]. Different age groups correspond to different FeNO detection value domains, with the asthma criterion for children being FeNO > 35 ppb and the criterion for adults being FeNO > 50 ppb [171]. Exhaled H_2_S concentrations correlate with the type of airway inflammation. The range of exhaled H_2_S concentrations detected by electrochemical sensors in patients with eosinophilic and granulocytopenic asthma is much lower than in healthy individuals. It ranges from 7.70 ± 4.20 ppb to 11.1 ± 4.60 ppb compared to 26.9 ± 4.60 ppb in healthy individuals [172,173]. The Jing et al. team [126] has developed an ultrasensitive chemoresistive H_2_S gas sensor based on a γ-Bi_2_MoO_6_-CuO heterostructure. The sensor used a heterostructured material formed by modifying p-type CuO nanoparticles on the surface of spherical, n-type, γ-Bi_2_MoO_6_ microspheres. By using first-principles calculations, the stable adsorption and charge transfer mechanism of H_2_S on the CuO surface was revealed, fully explaining its ultra-sensitivity with a detection limit of 5 ppb at 180 °C. Through clinical experiments, the study tested exhaled breath samples from 28 asthmatic patients and 28 healthy individuals (in Figure 5c for the sensor’s dynamic monitoring mode and Figure 5d for the specific detection scheme). According to the study, asthmatic patients had substantially lower H_2_S concentrations than healthy people. In addition, during the treatment process, the relationship between the increase in H_2_S concentration and disease remission can be dynamically tracked by sensors. The sensor’s integrated circuit can be developed into a portable device with stability and great selectivity (except from interference from acetone and NO). Particularly for pediatric and elderly patients, this offers a low-cost option for noninvasive asthma diagnosis and daily monitoring. Increased ROS, lipid peroxidation, the production of alkanes and aldehydes, and VOCs are all consequences of chronic asthma inflammation. The metabolic activity of inflammatory cells, such as neutrophils and eosinophils, can release these VOCs [165]. Machine learning-assisted gas sensing technology provides a safer, more convenient means of monitoring asthma management by analyzing inflammatory markers in exhaled breath [174]. Nidheesh et al. [127] have used a multi-wavelength UV photoacoustic spectroscopy (PAS) sensor (in Figure 5e) coupled with a 266 nm laser excitation source to detect VOCs in exhaled breath for asthma and COPD diagnosis. The sensor system detected the acoustic wave signals generated by VOCs absorbing laser energy through a photoacoustic cell and a lock-in amplifier. PCA is used for categorization and downscaling of the data. Four distinct laser wavelengths were used to compare the mean PAS signals of healthy and sick samples. The Cyranose-320, a commercial electronic nose with an array of 32 chemical sensors that use multi-sensor response patterns to determine illness features, was also used in the study as a comparison. The study employed a “match/mismatch” testing strategy in addition to PCA to classify data by combining spectral residuals and Mahalanobis distance. Additionally, the study used receiver operating characteristic (ROC) curves and the area under the ROC curve (AUC-ROC) to evaluate diagnostic signals. The diagnostic performance was assessed using the AUC-ROC in Figure 5f. There were 25 normal samples and 24 samples with asthma in the experimental set. Each sample underwent five repetitions of the measurements, yielding a total of 245 datasets. With an AUC-ROC of 0.948, the PAS sensor demonstrated 88% sensitivity and 89% specificity at 266 nm excitation. This was better than the 85.71% sensitivity and 84% specificity of the electronic nose. The investigation is limited by its small sample size, though, and it will be interesting to see if the findings hold up when the sample size is increased further.

### 4.2. Metabolism and Nutrition Disorders

The incidence of metabolic diseases, such as diabetes and obesity, continues to rise. Traditional testing methods, such as blood and urine tests, kidney biopsies, ultrasounds, CT scans, and MRIs, are invasive and uncomfortable for patients. They are also time-consuming and expensive. Some require specific environments and specialized personnel, which limits their application.

Prolonged hyperglycemia in diabetes mellitus can cause irreversible damage, including cardiovascular and cerebrovascular lesions, renal failure, blindness, and amputation [175], which seriously threatens quality of life. Early screening can effectively control blood glucose fluctuations through early intervention, significantly reducing the risk of complications and maximizing protection of organ function to prolong healthy survival. However, diabetes detection technology faces an escalating contradiction between the efficacy bottleneck of traditional methods and clinical needs. Current mainstream testing methods, such as the fingertip glucose meter, the glycosylated hemoglobin (HbA1c) test, and the oral glucose tolerance test (OGTT), have obvious shortcomings. Invasive blood collection decreases patient compliance, single-point measurements do not reflect dynamic metabolic profiles, and laboratory testing is time-consuming and costly. Known diabetes markers include VOCs, such as acetone, ethylbenzene, xylene, toluene, ethane, pentane, isoprene, isopropanol, 2,3,4-trimethylhexane, tridecane, ethanol, and methanol [176,177,178]. Acetone metabolism in the body is primarily divided into two processes. First, acetyl coenzyme A, which is generated from fatty acid oxidation, can be further metabolized in the liver to produce acetone [179,180,181]. Second, 2-propanol is converted to acetone by hepatic ethanol dehydrogenase (ADH) [178]. Acetone is a natural metabolic intermediate of lipolysis and a potential biomarker for monitoring diabetes and measuring glucose levels [179]. Elevated acetone levels, however, indicate insufficient insulin in the cells or inefficient insulin utilization [181]. Normal respiratory acetone levels range from 0.3 to 2.5 ppm. Once the exhaled concentration exceeds 3 ppm, it may indicate metabolic derangement [182,183]. The exhaled concentration ranges from 2.2 to 21 ppm in patients with type 1 diabetes mellitus and from 1.76 to 9.4 ppm in patients with type 2 diabetes mellitus [184,185,186,187]. Diabetic patients had acetone exhalation levels greater than 1 ppmv, HbA1c levels of at least 9.7%, and blood glucose levels ranging from 76 to 240 mg/dL. These levels were all significantly above normal. Healthy individuals had acetone exhalation, HbA1c, and blood glucose levels within the normal range [186]. Thus, it can be seen that acetone concentration trends are consistent with clinical gold standard levels, such as HbA1c and blood glucose. Gas sensors using novel nanomaterials, such as MXene (Ti_3_C_2_T_x_) and MoS_2_ composites [188], have achieved ppb-level detection sensitivity. The cost of individual sensors has dramatically decreased due to the low sales of wearable devices.

**Figure 6:**
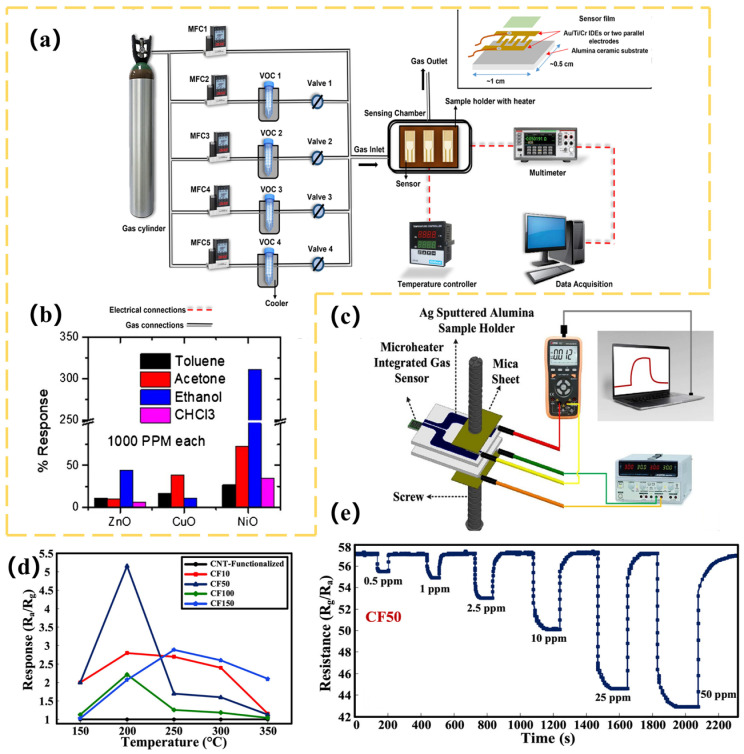
(**a**) Schematic diagram of the gas sensing device used in the experiment, including components such as the sensor array, gas path control system (mass flow controller), heating sample stage, and electrochemical workstation. (**b**) Comparison of the responses of three sensors to gases with a concentration of 1000 ppm [129]. (**c**) Gas sensing test device. (**d**) Response curves of nanocomposite sensors with different α-Fe_2_O_3_ contents to 10 ppm acetone within a temperature range of 150–300 °C. (**e**) Dynamic resistance curve of the CF50 sensor to 0.5–50 ppm acetone at 200 °C [130].

**Figure 7:**
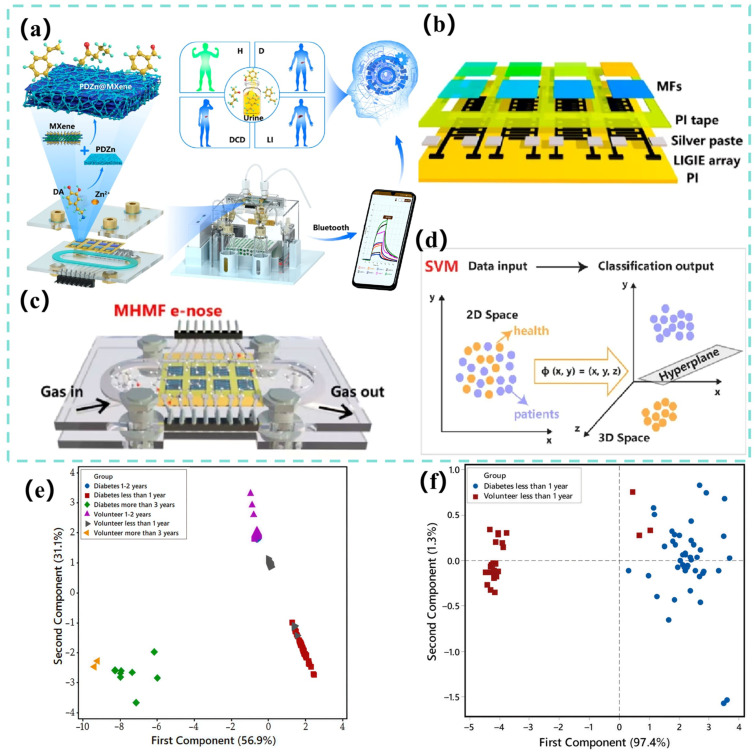
(**a**) Construction of MHMF electronic nose and portable point-of-care testing (POCT) platform, achieving urine VOC monitoring and non-invasive disease diagnosis through machine learning (ML). (**b**) Configuration of the MFs sensor array. (**c**) Assembly diagram of MHMF electronic nose. (**d**) The principle of the SVM algorithm for classifying health and mapping patients from low-dimensional to high-dimensional spaces [131]. (**e**) The PCA analysis scatter plot of the 0–4 year sample shows that the diabetes samples stored for 0–4 years overlap seriously with the healthy samples and have low discrimination. (**f**) PCA analysis of samples stored for less than one year indicated that among the samples stored for less than one year, the diabetes group was significantly separated from the healthy group [132].

The gas sensor array captures composite VOCs in exhaled breath. The machine learning network then decouples environmental disturbances from biomarker signals through feature engineering to form an acetone–blood glucose correlation model [189,190,191]. Zhu et al. [128] have utilized an array of seven MOS sensors (TGS822, TGS2620, WSP2110, MP503, MQ3, MQ135, and MP4) to collect 62 sets of acetone and ethanol gas data. After preprocessing, they used KPCA for nonlinear feature extraction and AdaBoost combined with a decision tree weak classifier for qualitative identification of gases. They automatically optimized the number of classifiers using grid search (GS). They used a multivariate correlation vector machine (MVRVM) for quantitative prediction of gas concentration. The results showed that the hybrid model achieved 99.722% accuracy in the qualitative identification of acetone and ethanol with a root mean square error (RMSE) of 0.027 and 0.030 ppm, respectively, and 94.55% classification accuracy on the gas sensor drift dataset with an RMSE of 11.59 and 8.72 ppm for acetone and ethanol, respectively, which is significantly better than the SVM and XGBoost algorithms. Singh’s team [129] has used three MOS sensors—NiO-Au (ohmic contact), CuO-Au (Schottky contact), and ZnO-Au (Schottky contact)—to construct gas sensor arrays. The device diagram is shown in Figure 6a. The sensors were prepared using DC reactive magnetron sputtering. The NiO and CuO films have rough, ultrafine-grained surfaces, and the ZnO film consists of nanosheets. Cross-sensitivity and machine learning algorithms were used to design the array for detecting four volatile organic compounds (ethanol, acetone, toluene, and chloroform) in a complex mixture of gases (in Figure 6b for results). Nine machine learning models (RF, KNN, decision tree, linear regression, logistic regression, plain Bayes, LDA, ANN, and SVM) were used in the study. The dataset contained single-gas (2,241,436 samples), two-gas (227,617 samples), and three-gas (131,120 samples) mixtures. After preprocessing, the dataset was divided into training, validation, and test sets. The results show that KNN and RF have more than 99% accuracy in classification, the *R*^2^ value of KNN regression analysis is greater than 0.99, and the acetone detection limit is as low as 0.012 ppm. The array, when combined with machine learning algorithms, can efficiently classify and quantitatively predict the composition of complex gas mixtures. This overcomes the lack of selectivity limitation of traditional MOS sensors. However, the above examples use synthesized gases, not disease models. The applicability of these models to more complex VOCs exhaled by diseased populations remains to be verified with real samples. Ansari et al. [130] have developed a gas sensor based on an α-Fe_2_O_3_-MWCNT nanocomposite. The nanocomposite CF50 material of α-Fe_2_O_3_ and multi-walled carbon nanotubes (MWCNT) was adopted as the core of the sensor. The nanocomposite was optimized through acid treatment and chemical synthesis to produce CF50 as the optimal sensing material (in Figure 6c–e). Integrating a micro-heater, temperature and humidity sensor, and platinum (Pt) interdigital electrode, the device was constructed as a portable handheld device. At the same time, 8-s rapid preheating and real-time LCD display were also supported. A 17-layer CNN combined with the Adam optimizer was used to train exhaled breath data from 50 volunteers; 45 of the volunteers’ data were used for model construction. Experiments showed that the optimized sensor exhibited good linearity in response to 5.15–10 ppm of acetone at 200 °C. It maintained a response value of 3.7 in a high-humidity environment of 82% and demonstrated excellent selectivity for interfering gases, such as CO_2_ and NH_3_. After calibration, the device achieved 85% accuracy within the blood glucose detection error of ±15 mmol/L with a response time of only five seconds, demonstrating good long-term stability. This intelligent sensing system is expected to subsequently connect to a smartphone for real-time analysis through a cloud-learning, continuous optimization model. Ultimately, it will be built into a personalized metabolic monitoring and early warning intervention management system. Ding et al. [131] have constructed a sensor array using a library of gas-sensitive materials based on porous MXene frameworks (MF) (in Figure 7a). The array consisted of eight modularly assembled, ligand-driven MFs (PDZn@MXene, PDCo@MXene, PDCa@MXene, and PDZnOA@MXene, as well as other metal-ion-doped and ligand-engineered variants) that serve as sensing units. These units were integrated onto a laser-induced graphene (LIG) fork-finger electrode array and encapsulated in a 5.44 × 4.50 × 0.42 cm^3^ laser-engraved microchamber. This formed a microchamber-bearing MF e-nose (MHMF e-nose) (in Figure 7b,c). The VOC fingerprints of clinical urine samples were analyzed using a SVM machine learning algorithm to diagnose 30 healthy people, 20 depressed patients with diabetes comorbidities, and 50 diabetic patients. SVM achieved an overall accuracy of 91.7% in the classification task (Figure 7d shows the classification principle). SVM’s performance was significantly better than KNN (85.6%), RF (83.3%), and ANN (79.2%). SVM achieved an area under the ROC curve of 0.98, a sensitivity of 88.9%, and a specificity of 96.8%. These results enabled real-time wireless monitoring of urinary VOCs and provided an efficient tool for noninvasive disease diagnosis. Esfahani [132] et al. have analyzed urinary VOCs using two electronic nose systems, FAIMS and FOX 4000. Among them, the field asymmetric ion mobility spectrometer (FAIMS), which is itself a chemical separation detector based on the difference in mobility of ions in a high electric field, generates complex ion mobility spectra by scanning dispersive fields and compensating voltages (−6 V to +6 V) containing 52,224. The FOX 4000 electronic nose (produced in Toulouse, France and purchased from Alpha M.O.S) was constructed with an array of 18 MOS gas sensors with different response characteristics for oxidizing gases, NH_3_, ethanol, organic amines, H_2_S, hydrocarbons, organic solvents, aromatic compounds, chlorinated compounds, aldehydes, etc. The response was determined by calculating the rate of change in the sensor resistance ((R_0_ − R_t_)/R_0_) to quantify it. The research team collected and analyzed 140 urine samples (73 patients with type 2 diabetes, 67 HC), which were frozen at −80°C within 2 h of collection and stored over a span of 4 years. The FAIMS data were first preprocessed and feature-compressed by applying a two-dimensional wavelet transform (Daubechies D4), then combined with a Wilcoxon rank sum test. Supervised feature selection was performed, and PCA was utilized to observe the data distribution (the PCA results were shown in Figure 7e,f). For the FOX 4000 data, the sensor maximum resistance variance was extracted as a feature, and the Boruta feature selection package was used. Ultimately, four machine learning classification algorithms, sparse logistic regression, RF, Gaussian process, and SVM, were used to construct the diagnostic model, the model parameters were tuned by validation set to prevent overfitting, and the performance was evaluated by an independent test set and ROC. The results showed that FAIMS performed best when analyzing urine samples stored for up to 1 year, with an AUC of 94%, sensitivity of 92%, specificity of 100%, and an AUC of 88% even when all samples aged 0–4 years were included. The sparse logistic regression model of the FOX 4000 achieved excellent AUC of 99%, sensitivity of 98%, and specificity of 97% when analyzing samples stored for up to 18 months; and the sparse logistic regression model analyzing samples aged 0–4 years achieved excellent results of AUC of 99%, sensitivity of 98%, specificity of 97%, and an optimal AUC of 89% when analyzing all samples from 0–4 years. This study not only demonstrated the feasibility of the two electronic noses to differentiate diabetes from healthy people using urine VOCs with high accuracy but also clearly indicated that storage of urine samples for more than 12–18 months significantly reduces the abundance and diagnostic accuracy of VOCs, which provides an important experimental basis and technological pathway for the development of a low-cost, noninvasive, and rapid diagnostic tool for diabetes mellitus.

### 4.3. Hepatobiliary Disorders

Modern methods of monitoring hepatobiliary disorders, such as hepatocellular carcinoma (HCC), have significant limitations. Conventional tests include endoscopy, blood biochemical analysis, stool testing, ultrasound, CT/MRI imaging, and tissue biopsy. These tests are invasive, some requiring an empty stomach or anesthesia. They also require complex procedures, long waiting times, and high costs. An accurate diagnosis depends heavily on specialized equipment and the technical expertise of the operator. This makes it difficult to popularize in primary medical institutions or resource-poor areas, resulting in insufficient early screening coverage.

**Figure 8:**
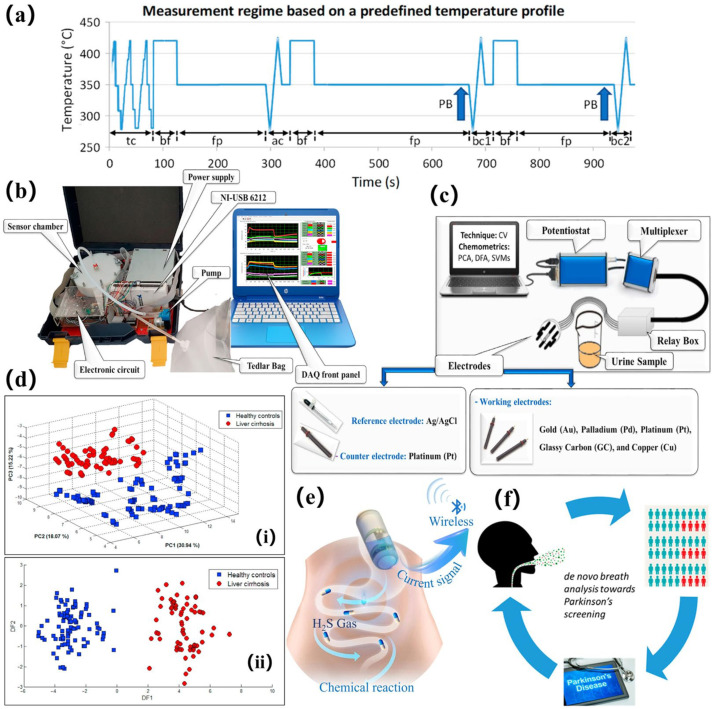
(**a**) Schematic diagram of measurement protocol based on sensor heater temperature control [133]. (**b**) Schematic diagram of the electronic nose system architecture. (**c**) Schematic diagram of the electronic tongue system architecture. (**d**) Data fusion PCA (**i**) and DFA (**ii**) results, blue area = HC, red area = LCI [135]. (**e**) Overall structure and working principle of the swallowable capsule platform. (**f**) Screening model for Parkinson’s disease based on breath analysis [139].

According to WHO data, liver cancer is the third deadliest cancer worldwide. Current liver cancer detection technologies face challenges regarding sensitivity and accessibility. Each year, there are more than 900,000 new cases of liver cancer, 70% of which have progressed to intermediate or advanced stages by the time of diagnosis, which highlights the limitations of traditional detection methods for early screening. Cirrhosis is a chronic, irreversible liver disease. Its main hazards include causing liver failure and an elevated risk of HCC. Cirrhosis can lead to serious complications, such as variceal bleeding or infection [192]. Serum marker tests for diagnosing HCC, such as serum alpha-fetoprotein (AFP) [193], have low overall sensitivity, ranging from 0.32 to 0.61. The higher the sensitivity, the lower the threshold for AFP detection of HCC. Imaging has limited recognition of early microscopic lesions, and liver biopsy carries a risk of trauma. Cirrhosis is primarily diagnosed through a combination of medical history, symptoms, blood tests, imaging, and liver biopsies. More efficient and rapid methods are lacking. In recent years, studies have shown the potential of electronic noses for the early detection of liver disease. Of the more than 3500 VOCs found in human exhaled gas, 1-octanol, 3-hydroxy-2-butanone, styrene, decane, and hexanal [194,195] have been identified as biomarkers of specific metabolism in patients with HCC, and their concentrations exhibit significant, characteristic changes. On the other hand, significant gas markers of cirrhosis were dimethyl sulfide, limonene, acetone, 2-butanone, 2-pentanone, indole, and dimethyl selenide [195]. The synergistic working mechanism of machine learning models and gas sensors has created a new paradigm for liver disease screening. The system collects multidimensional gas signals through sensor arrays and, after preprocessing, constructs dynamic VOC fingerprints or intensity chromatograms. Voss et al. [133] have used a wearable electronic nose system based on MOS sensors to detect liver dysfunction. The system contained three sensor modules (S1, S2, and S3), each of which integrates a different metal oxide-sensitive layer. S1 uses a catalyst-doped SnO_2_-thick film layer that mainly detects CO, H_2_, and ethanol. S2 and S3 contain multilayered structures of SnO_2_ and WO_3_, respectively, that detect reducing gases, gases that can easily be oxidized, and long-chain hydrocarbons. The sensor array optimizes gas response characteristics through temperature modulation, building multidimensional detection capabilities with nine sensitive layers (in Figure 8a). Ten time-domain and nonlinear kinetic features were extracted from the respiratory gas measurement cycle and classified using a combination of LDA and leave-one-out cross-validation. The study tested 30 subjects (10 with HC, 10 with compensated cirrhosis, and 10 with decompensated cirrhosis). The system demonstrated 100% sensitivity, specificity, and accuracy in distinguishing healthy individuals from cirrhotic patients, and 95% accuracy in distinguishing between compensated and decompensated cirrhosis. This system achieved near-perfect classification performance in cirrhosis staging detection and can be extended to liver cancer detection based on different VOCs. This system’s ability to rapidly and noninvasively assess liver function impairment and staging is significant. Nazir et al. [134] have constructed a modified electrode array based on glassy carbon electrode (GCE) for the detection of 2,2′-methylenebis(6-tert-butyl-p-cresol) (MBMBP) in the breath of patients with HCC using electrochemical sensors with hexanethiol monolayer-modified gold nanoparticles (AuNPs) as the core sensing material. The study first screened breath samples from 35 HCC patients and 30 HC patients by GC-MS combined with unsupervised machine learning models (PCA and heat map analysis), in which MBMBP was identified as a significant biomarker of HCC because of its lowest concentration of 2100 ppm and frequency of occurrence of 87.6% in the patients. Subsequently, the team synthesized hexanethiol-capped AuNPs and modified them on the surface of the electrode, utilizing their electrochemical redox reaction with MBMBP to transfer 2e^−^/2H^+^ electrons and form phenol–oxygen radicals for specific detection. The sensor demonstrated high sensitivity to MBMBP by electrochemical techniques such as differential pulse voltammetry (DPV) under the optimized conditions of pH 4.0 and a scan rate of 100 mV s^−1^, with an LOD of 0.005 mol L^−1^ and a linear range of 0.15–0.38 M, and its validity was successfully validated in clinical respiratory samples, providing a high-potential portable platform for the noninvasive diagnosis of early HCC. Zaim’s team [135] has constructed a multi-sensor system using an electronic nose and voltammetric electronic tongue for differentiating patients with liver cirrhosis (LC) from HC. The electronic nose contained two types of sensor arrays, five commercially available chemical gas sensors (MQ-2, MQ-3, MQ-135, MQ-137, MQ-138) and six fork-finger gas sensors based on pristine or metal-doped WO_3_ nanowires (including pristine WO_3_, Pt/WO_3_, Au/Pt/WO_3_, Au/WO_3_, Ni/WO_3_, Fe/WO_3_), the latter operating at specific temperatures of 100 °C or 160 °C and at UV 394 nm (in Figure 8b). For the electronic tongue, a five-electrode voltammetric array (copper, glassy carbon, platinum, palladium, and gold working electrodes) was used to analyze urinary VOCs by cyclic voltammetry (in Figure 8c). A total of 54 volunteers (22 LCI patients, 32 HC) were recruited for the study, and 3 breath samples per person, totaling 162 and urine samples with six repeated measurements per sample, were collected to extract sensor response characteristics. Data were analyzed using PCA, discriminant function analysis (DFA), SVM, and ROC curves. The classification effect was limited when using e-nose or e-tongue alone, with PCA/DFA showing sample overlap, e-nose ROC-AUC = 0.965, and e-tongue ROC-AUC = 0.950; SVM classification accuracies were 98.33% and 97.50%, respectively. In order to improve the performance, a mid-level data fusion strategy is proposed, i.e., merging the feature data of two devices—ΔG/AUC of electronic nose and Ptox/Area of electronic tongue. After fusion, the model is significantly optimized, with PCA clearly distinguishing between the two types of samples, and the DFA achieving complete separation (in Figure 8d), with the ROC-AUC reaching 0.999 and the SVM classification accuracy being improved to 100%. The results demonstrated that the combined analysis of breath and urine VOCs could realize the non-invasive screening of LC through multi-sensor fusion, which provided a new method for early diagnosis.

### 4.4. Gastrointestinal Disorders

Gastrointestinal disorders are diverse and complex, with common symptoms including postprandial fullness, belching, nausea and vomiting, abdominal pain, and abnormal bowel movements (such as diarrhea, constipation, or mucus in the stool). In severe cases, symptoms of gastrointestinal bleeding, such as hematemesis or melena, may occur. Long-term gastrointestinal dysfunction may also lead to systemic issues such as malnutrition, anemia, and weight loss [196,197,198]. If these diseases are not treated promptly, they may develop into chronic inflammation, ulcers, or even cancer, such as gastric cancer or intestinal cancer. Therefore, early diagnosis is crucial for improving treatment outcomes, but traditional detection methods have many limitations. Wilson [199] pointed out that invasive examinations such as colonoscopy, tissue biopsy, and microbial swab culture not only cause discomfort to patients but are also limited by equipment costs and professional operational requirements. Gas sensing technology provides a more convenient solution for screening such diseases by analyzing VOCs released in exhaled breath or from the body surface [200].

Currently, the causes of gastrointestinal diseases can be mainly divided into infectious pathogens, host metabolic abnormalities, and intestinal flora imbalance. The VOC gas markers produced by different causes are not the same. The gas markers for gastric ulcers caused by Helicobacter pylori infection are 2-butanone, 1-propanol, and CS_2_; for Salmonella infection, they are acetonitrile and ethanol; for Shigella infection, they are 2-propanol; and for enterovirus infection, they are ethyl dodecanoate and propyl dodecanoate [199,201]. These are mostly metabolic byproducts of bacteria, viruses, or parasites in the human body. Abnormal metabolic gas markers include H_2_S, 1-octene, 1-decene, (E)-2-nonene, esters, indole, NO, methane, acetaldehyde, terpenes, benzene ring compounds, 4-methylheptane, 4-methyloctane, and so on [199,202]. Due to disease-induced alterations in host cell metabolic pathways, specific VOCs are produced. In patients with inflammatory bowel disease (IBD), activated immune cells release ROS, leading to oxidative stress and the generation of short-chain aldehydes and nitrogen-containing oxides. In gastrointestinal cancer patients, cancer cells enhance glycolysis through the Warburg effect, producing ketone bodies and branched-chain fatty acid derivatives. Dysbiosis of the gut microbiota also produces corresponding gas biomarkers. A reduction in Bifidobacterium leads to decreased levels of short-chain fatty acids such as acetate and butyrate, which are associated with inflammation. An increase in Enterobacteriaceae leads to the fermentation of proteins to produce indole and sulfides [201]. These VOCs originate from gases emitted in exhaled breath, feces, and urine. Stine et al. [203] have developed a swallowable capsule system for the in situ detection of H_2_S in the gastrointestinal tract, measuring 14 mm × 34 mm. The core of the system employed a three-electrode electrochemical array sensor based on Au electrodes, comprising a gold working electrode, a gold counter electrode, and a silver reference electrode (in Figure 8e). The sensor innovatively integrated a perfluorosulfonic acid resin (Nafion) nano-film as a solid-state electrolyte, which was optimized for conductivity and moisture retention through 1 M sulfuric acid pretreatment. The surface was covered with a polyethylene tetrafluoride (Teflon) breathable membrane to achieve gas–liquid separation. Sensor performance validation was conducted in a custom gas testing apparatus, with a sample set covering physiologically relevant concentrations (0.9–9 ppm H_2_S). The amperometric method was employed with a +0.1 V bias voltage, enabling direct quantification via current response. The results indicated that the sensor exhibits excellent linear response and high selectivity in humid environments (H_2_S/H_2_ response ratio of 5.69, far exceeding the 1.43 of commercial SPEC-H_2_S sensors) (in Figure 8f). The capsule prototype successfully achieved wireless real-time monitoring of trace H_2_S at 1 ppm and 3 ppm, with current responses of ~79 nA and ~99 nA, respectively. This work marks the first construction of a miniaturized H_2_S sensing platform suitable for the complex gastrointestinal environment, providing a breakthrough tool for studying the association between intestinal gas dynamics and disease. However, the long-term stability of the sensor still requires further optimization to suppress drift. The sensor array integrated with machine learning performs non-invasive diagnosis based on the VOCs described above. Tyagi et al. [136] have used a portable electronic nose (PEN3) equipped with an array of 10 MOS sensors (W1C, W5S, W3C, W6S, W5C, W1S, W1W, W2S, W2W, W3S), combined with gas chromatography time-of-flight mass spectrometry (GC-TOF-MS) technology, to diagnose colorectal cancer (CRC). The sample set included 96 urine samples (58 CRC patients, 24 in the early stage and 34 in the late stage, and 38 non-cancer controls). The PEN3 sensor is based on traditional metal oxide technology, with an operating temperature range of 250–550 °C, capable of detecting various substances such as aromatic compounds, hydrogen, and sulfides. The algorithm employs RF and neural network models, with data analyzed via 10-fold cross-validation. Among these, the neural network demonstrated the best performance in distinguishing between CRC and non-cancer groups, achieving an AUC of 0.81. This combined approach identified 23 potential CRC biomarkers among VOCs, including octanal and nonanal, with 11 of these validated by literature review. While the method effectively distinguishes between cancer stages and non-cancer groups, its ability to differentiate between early and advanced stages is limited. This approach offers a new, low-cost, non-invasive screening strategy for colorectal cancer in clinical settings. Arasaradnam et al. [137] have used an electronic nose (Fox 4000) equipped with 18 MOS sensors and a field-asymmetric ion mobility spectrometer (FAIMS) to analyze VOCs produced in the urine of IBD patients. The study included 62 participants (48 IBD patients, including 24 Crohn’s disease (CD) patients and 24 ulcerative colitis (UC) patients, divided into relapse and remission groups, and 14 HC patients). The electronic nose utilized the sensor array to respond to gas-formed “odor fingerprints” combined with PCA and DFA for data dimensionality reduction and classification. FAIMS employed a high-voltage electric field to separate ionized molecules, using wavelet transformation combined with Fisher discriminant analysis (FDA) to optimize classification. The results showed that both techniques achieved an accuracy rate exceeding 75% (*p* < 0.001) in distinguishing IBD patients from healthy individuals and could effectively identify disease activity status in subgroup analyses. This study validated the association between VOC-based “fermentation group” characteristics and gut microbiota dysbiosis, providing a new approach for non-invasive IBD diagnosis and dynamic monitoring. Further validation with a larger sample size is recommended.

### 4.5. Nervous System Disorders

Early and accurate detection of neurological diseases has always been an important challenge for the medical community. The diagnosis of neurodegenerative diseases such as Alzheimer’s disease (AD) and Parkinson’s disease (PD) is highly dependent on imaging, cerebrospinal fluid puncture analysis, and neuropsychological scale assessment. These traditional methods are limited by the risk of radiation exposure, invasive trauma, subjective judgment bias, and high testing costs. Especially for early-stage patients with weak biomarker concentrations and insidious symptoms, the sensitivity of conventional methods is insufficient, resulting in the diagnosis being made in the middle to late stages of the disease. Gas sensor technology opens a new path for early screening of neurological diseases. Recent studies have revealed that there is a significant association between VOCs in human exhaled gas and neurological disorders, such as abnormally high concentrations of NO, benzaldehyde [204], nonanal, and 6-methylnonanone in the exhaled breath of AD patients and characteristic fluctuations in dopamine (DA), ascorbic acid (AA) [205], 1-butanol, and 2-methylfuran [138] in PD patients. When exposed to the patient’s exhaled gas or body fluid samples volatilized gas, the sensor produces physical or chemical responses such as conductivity and resistance due to changes in gas composition, forming a multidimensional “odor fingerprint”.

A deep synergy between machine learning models and gas sensors can create systems that range from data collection to intelligent diagnosis. Bach’s team [138] has used a Cyranose C-320 handheld device with a built-in array of 32 carbon black–polymer composite nanosensors. During the test, subjects inhaled medical-standard air and then continuously exhaled at a flow rate of 100–200 mL/s for ten seconds into a collection bag. Measurements were repeated three times for each set of samples and normalized to the median. The sensing array generates a unique, 32-dimensional “odor fingerprint” signal by absorbing the VOCs in exhaled breath, which causes a change in electrical resistance. This change in resistance causes the polymer to expand or contract, thereby changing the spacing of the carbon black particles. Using PCA downscaling and LDA classification algorithms, three groups were differentiated significantly in 74 samples (39 AD, 16 PD, and 19 HC) (*p* < 0.0001 between groups), with 50–77% sensitivity and 68–77% specificity in cross-center validation. The Finberg research team [139] has used a nanomaterial-based sensor array to detect VOCs in exhaled breath via 40 cross-reactive sensors as shown in Figure 8f. The sensor core materials consisted of two types, gold nanoparticles (GNPs) and walled carbon nanotubes (CNTs), both of which were functionalized and modified by different organic chemical ligands to enhance sensitivity to specific VOCs. The sensor arrays generated data by measuring the electrical resistance changes caused by the interaction of VOCs with the nanomaterial films; four feature values were extracted for each sensor (electrical resistance change in the middle/end of the signal, peak response, and AUC), and the optimal features were ultimately determined by feature filtering for modeling. DFA, a supervised pattern recognition method, was used to construct a linear classification model by maximizing the between-group differences and minimizing the within-group differences, and the leave-one-out method was applied to cross-validate the assessment performance. The current sample consisted of 29 patients with primary unmedicated PD 19 HC. The sample was rigorously screened (excluding other neurological disorders and drug interference), and the grouping was confirmed by auxiliary diagnostics such as midbrain ultrasound (TCS), olfactory sensory test (UPSIT), and non-motor symptom questionnaire (NMSQ). The final sensor array performance for PD detection was 79% sensitivity, 84% specificity, and 81% accuracy (86% area under the ROC curve). The performance was superior to the olfactory test (73% accuracy), but slightly lower than midbrain ultrasound (92% accuracy). The study demonstrates for the first time that the technique is effective in unmedicated early-stage PD patients and is not interfered with by therapeutic drugs, providing a basis for the development of a portable screening tool. The sensitivity can be further improved in the future by optimizing the transducer design and sampling process.

### 4.6. Renal and Urinary Disorders

The kidneys and bladder, core urinary system organs, handle waste excretion, electrolyte balance, and internal environment stability. However, current urinary tract disease diagnostics have critical limitations, as follows: invasive biopsies carry the risks of bleeding or infection and have a low acceptance rate among patients; key renal function blood markers (e.g., creatinine) lack sensitivity until severe impairment, hindering early warning; invasive cystoscopy for bladder cancer screening is painful, while non-invasive urine cytology poorly detects early tumors and risks underdiagnosis. These flaws in invasiveness, sensitivity, and dynamic monitoring impede early detection, timely intervention, and grassroots screening of urologic diseases.

#### 4.6.1. Nephropathy

The kidneys are an important “filter” of the human body. They bear the heavy responsibility of excreting waste and regulating electrolyte balance. However, chronic kidney disease (CKD) has an insidious onset with no obvious symptoms in the early stages. According to the WHO’s global health estimates, CKD accounted for 1.5% of global deaths in 2012. Currently, CKD affects about 7–12% of the population in different regions of the world, totaling 850 million patients [141]. Diabetes mellitus and hypertension are major contributors to the development of CKD [142,206,207]. Glomerular filtration rate (GFR) is commonly used to test renal function. Normal values are approximately 90–120 mL/min/1.73 m^2^. Renal biopsy samples can show clear evidence of CKD, such as glomerulosclerosis, tubular atrophy, and interstitial fibrosis. The advent of gas sensors has opened new possibilities for diagnosing kidney disease. Common gaseous markers for the diagnosis of CKD include NH_3_, methylene chloride, 2-methyl-6-nitropyridine, 4-amino-1,2,4-triazole, styrene, and limonene [208,209]. A correlation was found between exhaled NH_3_ and blood urea nitrogen (BUN) and creatinine levels. Healthy individuals usually have breath NH_3_ concentrations below 29 ppb, whereas patients with end-stage renal failure can have mean breath NH_3_ concentrations ranging from 820 to 14,700 ppb. This makes breath NH_3_ analysis a promising means of monitoring renal disease [210]. Day et al. [140] have constructed an electronic nose array for thin gas detection using surface acoustic wave sensors combined with MOFs as sensing materials. They optimized a sensor array composed of five MOFs, taking full advantage of their high specific surface area and tunable adsorption properties to improve sensitivity to target gases. During the design process, combined linear adsorption coefficients (CLACs) were introduced to quantify the adsorption of trace gases and the displacement effect of background gases (nitrogen and oxygen) by the MOFs. The array performance was then optimized using the singular value decomposition (SVD) method. An iterative numerical model was also developed to invert the gas composition from the sensor output by progressively subdividing the gas composition space and incorporating probabilistic analysis. The experiment used 100 simplified simulated respiratory samples (50 healthy and 50 diseased) and successfully controlled the NH_3_ concentration detection error within the allowable range for clinical diagnosis. It also accurately differentiated between healthy (0.49 ± 0.08 ppm) and diseased (3.32 ± 2.19 ppm) samples [211], which validated the potential of this MOF array for the noninvasive detection of kidney disease. However, this experiment did not consider humidity interference or the complexity of actual breathing, and it only tentatively provides a feasible framework for multicomponent gas sensing.

**Figure 9:**
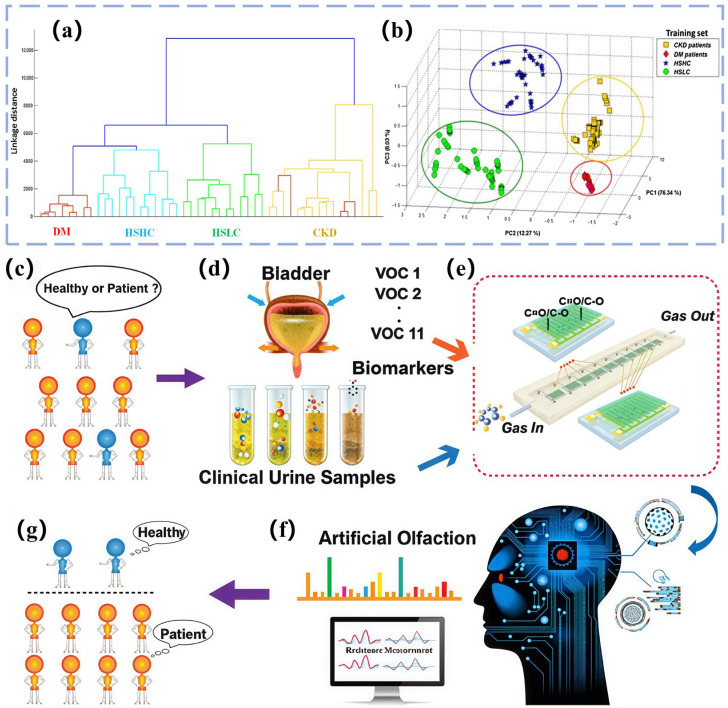
(**a**) HCA tree diagram, classifying exhaled gas samples according to their health status. (**b**) Three-dimensional PCA plot, showing the distribution of CKD, DM, HSHC, and HSLC samples [142]. (**c**) Collection of urine samples. (**d**) Biomarkers and clinical urine samples of BLC. (**e**) Electronic nose platform. (**f**) Artificial pattern recognition. (**g**) Results of pattern recognition [143].

Machine learning models can eliminate environmental noise using wavelet transforms. For example, they can attenuate acetone interference signals in breath by more than 90% [212]. A correlation model was used to integrate data such as exhaled breath composition, patient history, and physiological indicators to predict renal disease staging. Zhang’s team [141] has developed a wearable vision sensor based on a porous eutectic gel made of a nanocomposite material consisting of N-acryloyl glycine, glycerol, choline chloride, and bromocresol green (EG/BCG). A spiral-shaped porous structure (EG/BCG-spiral) was constructed using digital light processing 3D printing technology to significantly enhance the adsorption rate and specific surface area of NH_3_. The sensor efficiently enriches NH_3_ using the hydrogen bonding network inside the material, achieving a color change through BCG’s pH-responsive property. A CNN algorithm model was investigated to correlate color information (hue value) with BUN levels. Clinical data from 157 volunteers (training set: test set = 8:2) were used to train the model, which achieved 96.5% detection accuracy in the test set. The sensor exhibited a rapid response time, a detection limit of 0.5 ppm, a broad detection range spanning 0–10 ppm, and high stability under varying temperature and humidity conditions. It can accurately differentiate between healthy individuals and those with mild or severe CKD, as well as assess dialysis efficiency. This makes it a convenient tool for early screening and continuous monitoring of CKD. Saidi’s team [142] has used six commercially available MOS gas sensors (MQ-2, MQ-3, MQ-9, MQ-135, MQ-137, and MQ-138) to construct an electronic nose array. The array extracted features using dynamic conductivity slope (dG/dt), AUC, and conductivity change (ΔG). They used a combination of PCA, SVM, hierarchical cluster analysis (HCA), and partial least squares regression (PLS-Regression) algorithmic models to analyze 264 breath samples from 44 volunteers (16 patients with CKD, 6 diabetic patients, and 22 healthy individuals) in Figure 9a,b. The results showed that the SVM model achieved 100% accuracy in disease classification, PCA effectively differentiated between healthy and diseased populations, and the PLS model successfully established a quantitative relationship between breath characteristics and urinary creatinine levels (*R* = 0.91). These results validated the feasibility of using an electronic nose for the noninvasive diagnosis of CKD and provided a new, low-cost, portable method for clinical screening.

#### 4.6.2. Bladder Cancer

Bladder cancer [213,214] is one of the ten most common cancers worldwide. Given its high incidence rate and high recurrence rate, early detection and intervention are crucial. Advances in VOC biomarker detection and identification technologies have injected new vitality into the diagnosis of bladder cancer. Related studies have identified NH_3_, heptanal, hexanal, n-decane, 2-pentanone, toluene, and isoprene as biomarkers for bladder cancer [215]. As shown in Figure 9c–e, Jian et al. [143] have designed an artificial intelligence-enabled chemical resistive sensor array based on polyaniline (PANI) derivatives, utilizing algorithms such as SVM, PCA, and K-means to achieve a non-invasive, rapid, and highly accurate diagnosis of bladder cancer, suitable for early screening and post-surgical recurrence monitoring. As shown in Figure 9f,g, the sensor, combined with the PCA algorithm, demonstrates excellent classification performance for VOCs such as dimethyl disulfide, isopropyl alcohol, and hexanal, enabling effective identification of target VOCs. The SVM model was used as the main machine learning model in the study and was applied to the identification of sensor response data. With the assistance of algorithms such as K-means, high accuracy rates were achieved in both early diagnosis and postoperative monitoring. The experimental model demonstrated excellent diagnostic performance, as evidenced by an accuracy rate of 96.67% for the test set and an accuracy rate of 88.89% for postoperative recurrence monitoring, which was significantly better than traditional cystoscopy and urine cytology testing. Similarly, Huang et al. [144] have selected propanol, ethylbenzene, acetic acid, and NH_3_ as VOCs and constructed a sensor array using eight metal oxide gas sensors, including the TGS2600 and MP135. They then used AI technology to build a model and designed a bladder cancer detection system based on electronic nose technology, which greatly improved the convenience and accuracy of bladder cancer diagnosis. In this process, machine learning algorithms such as SVM, RF, KNN, and LDA were employed to achieve precise identification of VOCs biomarkers in the urine of bladder cancer patients, highlighting the system’s potential for clinical application. During model construction and evaluation, the single algorithms SVM, RF, and KNN classified 12-dimensional features with accuracies of 0.85, 0.56, and 0.79, respectively. Among them, RF performed the worst in classification, with a high rate of misdiagnosis of bladder cancer. The accuracy of SVM combined with PCA dimensionality reduction technology was 0.97, while that of RF combined with LDA reached 0.94. In contrast, LDA performed better in visualization analysis, effectively distinguishing between four VOCs, propanol, ethylbenzene, acetic acid, and NH_3_. KNN combined with LDA achieved 100% accuracy and recall for ethylbenzene, demonstrating perfect identification capability for this gas. Sensors play a unique and important role in all stages of bladder cancer detection, including early screening, diagnosis, and postoperative monitoring, providing strong support for precise diagnosis and treatment of bladder cancer. With further innovation in sensor technology, more benefits are expected to be brought to bladder cancer patients.

### 4.7. Reproductive System Disorders

Breast cancer [216,217,218] and prostate cancer are common reproductive system diseases, and early screening plays a crucial role in improving cure rates. Traditional methods for detecting reproductive system diseases include mammography [219] and prostate-specific antigen (PSA) testing [220]. Despite wide application, these methods carry inherent risks, including radiation exposure and elevated false positive rates. Breast cancer and prostate cancer can cause changes in metabolic products, and the characteristic volatile metabolites exhaled by patients are often used for disease diagnosis. In recent years, machine learning-assisted gas sensing technology has opened up new avenues for the non-invasive detection of these two cancers by analyzing VOCs released by the human body [204].

#### 4.7.1. Breast Cancer

A multitude of related studies have demonstrated that formaldehyde, pentane, hexane, long-chain alkanes, and alkane derivatives have been identified as biomarkers for breast cancer [221]. Gas sensors based on these biomarkers have brought about a new revolution in breast cancer detection [222], making high-precision, dynamic monitoring possible. Breath analysis [223] is a non-invasive, painless, and easily executable detection method. Breath analysis assisted by machine learning also significantly improves the efficiency of breast cancer detection. Yang et al. [145] have developed a new breath-based detection method for breast cancer, diagnosing the disease and its molecular subtypes by analyzing volatile metabolites in exhaled breath. This method uses an electronic nose consisting of 32 carbon nanotube sensors and employs eight machine learning algorithms, including k-nearest neighbor, naive Bayes, decision tree, neural network, support vector machine, logistic regression, gradient boosting, and random forests. Among these, the RF model demonstrated the best performance in disease detection. In testing, the method demonstrated high accuracy and reliability in predicting breast cancer, with an accuracy rate of 91%, sensitivity of 86%, specificity of 97%, positive predictive value of 97%, and negative predictive value of 97%. In addition, the RF model showed significant advantages in identifying molecular subtypes of breast cancer. Its average accuracy in leave-one-out cross-validation was 88.5 ± 12.1%, and its reliability coefficient was 0.77 ± 0.23.

#### 4.7.2. Prostate Cancer

**Figure 10:**
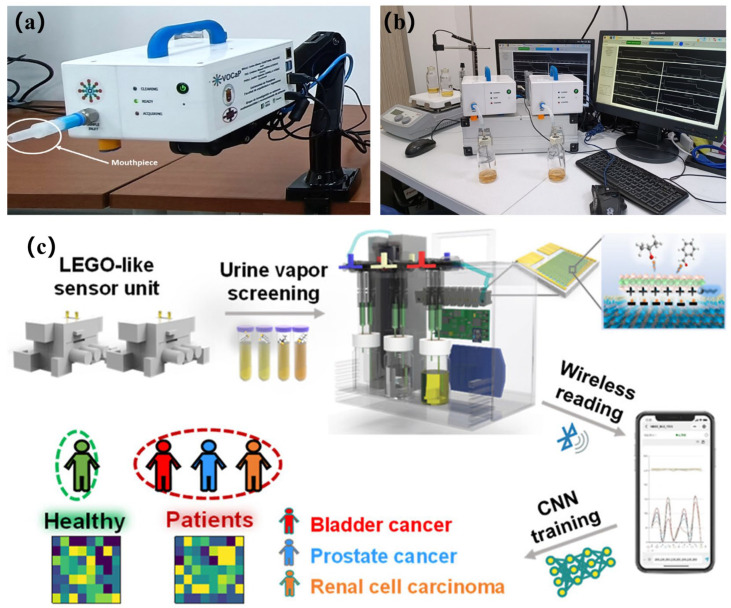
(**a**) Disposable mouthpiece device for collecting exhaled breath samples. (**b**) Sampling scheme for collecting urine to generate headspace. (**c**) Schematic diagram of the non-invasive detection platform for urinary tract diseases [146].

Machine learning-assisted gas sensors not only provide innovative ideas for breast cancer detection but also offer new directions for early clinical screening of prostate cancer. Gómez et al. [146] have improved the detection accuracy of prostate cancer by integrating data from electronic noses and electronic tongues. As shown in Figure 10a,b, MEMS gas sensor electronic noses were used to analyze patients’ exhaled gases and headspace volatile gases in urine, while carbon-based C110 and gold-based 250BT electrode electronic tongues were used to detect ions and metabolites in urine. Additionally, classification models were constructed using machine learning algorithms, such as PCA, SVM, k-nearest neighbors, and RF, and the performance differences between single-device data and fused data were compared. The results indicated that compared to analyzing VOCs with a single electronic nose or analyzing urine with a single electronic tongue, the fusion of multiple detection data enhances detection sensitivity, accuracy, and other aspects as shown in Figure 10c. After integrating exhaled gas and urine data using an electronic nose, the PCA decision model achieved 100% accuracy for all diagnostic indicators in prostate cancer patients compared to the control group. When using electronic nose and electronic tongue to integrate urine data, the DFA-SVM model achieved 98.4% sensitivity and 100% specificity for prostate cancer. The DFA-SVM models were applied to analyze the overall data of four groups of samples, prostate cancer, benign prostatic hyperplasia, prostatitis, and healthy individuals, achieving a classification accuracy rate of 97.3%. Among these, the sensitivity and specificity for prostate cancer reached 100% and 97.8%, respectively. Numerous studies have shown that data fusion technology, which combines gases and liquids and combines machine learning algorithms, is a highly promising method. These technologies can effectively improve the accuracy of early patient screening in clinical settings. Sun et al. [147] have developed a detection platform for non-invasive monitoring of urinary tract cancers, including bladder cancer, prostate cancer, and renal cell carcinoma. The platform used a chemical resistance sensor array composed of MXene-TMDC nanocomposites and drew inspiration from the construction method of Lego bricks. Algorithms such as CNN, PCA, KNN, and LDA were employed, with PCA and HCA used for data exploration, CNN responsible for classification, and traditional algorithms utilized for auxiliary validation. This formed a seamless workflow of “preprocessing–deep analysis–result validation,” providing an effective method for real-time cancer screening. The PCA exhibited a classification accuracy of 98% for 14 VOCs, while the CNN attained an accuracy rate of 90%, a sensitivity of 98%, and a specificity of 97% for the test samples.

## 5. Challenges and Development Directions

The use of gas sensors as a non-invasive detection method is expanding quickly. In the meantime, gas sensors are being used to achieve intelligence through the use of machine learning. The cutting-edge use of machine learning-assisted gas sensing technology in medical diagnosis is the main topic of this literature, which also methodically goes over the algorithmic synergistic mechanisms and working principles of electrochemical, optical, semiconductor, and other common uses of gas sensors in medical diagnosis, demonstrating their potential for use in multi-dimensional disease diagnosis. However, the aforementioned instances either lack real samples or have tiny sample sizes. Therefore, more study is required to confirm that electronic noses have a high potential for clinical diagnosis. In addition, the current technological development is facing multidimensional challenges, and the core issues can be analyzed from the dimensions of portability of sensor devices, high precision, real-time monitoring, and the development of new machine learning algorithms.

Portability and wearability are core factors that determine whether devices can be promoted significantly. Stringent power consumption requirements for wearable devices limit the use of traditional MOS sensors, which typically require an operating temperature of 200–400 °C [63] and a power consumption of approximately 140 mW [224]. This results in a device endurance of less than 24 h. Although electrochemical sensors have lower power consumption, their lifetime is limited by electrolyte volatilization. Additionally, issues such as the interfacial stability of flexible substrate materials and rigid sensing elements, temperature and humidity changes during long-term wear, and interference from the human body generating VOCs in different states (e.g., isoprene concentrations are significantly higher during exercise [225]) have yet to be fully resolved. Room temperature-sensitive materials (e.g., two-dimensional sulfide heterojunction) can significantly reduce detection power consumption and eliminate the need for heating [226]. The circuit design uses a pulse power supply and an intelligent triggering mechanism that activates full-power detection only when the gas concentration changes. Miniaturized sensor arrays based on MEMS/NEMS processes [107] combined with machine learning algorithms enhance anti-interference capability through multimodal data fusion. Breakthroughs in flexible electronics allow fully printed sensors to be integrated directly into fabrics. Devices can also be made with wireless communication modules (e.g., Bluetooth or NFC) through microfluidics, achieving passive detection and real-time data transmission to provide dynamic feedback for health management [227]. These improvements overcome the significant barriers of portability and wearability of sensor devices.

Sensors themselves can introduce errors such as baseline drift due to cross-sensitivity and environmental disturbances, signal coupling between sensors, and noise interference, which can be considered “noise.” If the amplitude of the signal representing changes in actual gas concentration is exceeded by the amplitude of noise, the machine learning model will struggle to learn effective patterns and instead fit the noise. Therefore, the accuracy of the sensor significantly impacts its integration with machine learning models. Additionally, high-precision sensor modes are more advantageous for extracting more effective, high-distinctiveness features and distinguishing between different gases with similar features. Most current gas sensors still suffer from low accuracy due to the aforementioned reasons. To address this, multiple sensors with different cross-sensitivity characteristics can be combined into a specific sensor array to generate multi-dimensional response signals, and various algorithms can be employed to enhance accuracy. PCA and LDA are widely used for data dimensionality reduction and visualization, enabling rapid gas category differentiation; SVM can handle nonlinear classification through kernel functions; and CNN is used to process time-series response signals, enabling dynamic gas identification, among other applications.

At present, due to the rapid development of machine learning algorithms, the market has put forward higher requirements for the functional innovation and performance breakthrough of gas sensors, especially in dynamic monitoring. On the one hand, by building structures such as cross-stacking [228], it is conducive to accelerating the response rate of sensors to gases and realizing millisecond breakthroughs. Machine learning, especially supervised and unsupervised learning, will realize efficient data analysis and promote the development of monitoring dynamics through the synergy of multiple algorithms. On the basis of massive medical gas sensor data learning, machine learning can mine the patterns and features behind the data to achieve the effect of accurate identification and rapid processing of disease gas features. For example, for diseases such as lung cancer [229] and diabetes mellitus [230], the mechanism of machine learning models working in concert with gas sensors will monitor patients’ exhaled gases in real time and speed up data processing. In the future, algorithms such as PCA and LDA will more effectively realize feature extraction and dimensionality reduction in the medical process. SVM, RF, ANN, and other algorithms will accurately classify diseases and distinguish between diseased and healthy samples with high accuracy. Models such as linear regression, SVR, and ANN regression will also help in medical disease prediction. With the addition of machine learning, various new technologies have been formed to add new opportunities to healthcare. For example, techniques such as machine learning and IoT have been used to construct wireless smart gas sensors. Accordingly, the bioelectronic mask constructed by Wang et al. [231] have fully embodied the characteristics of this technology. The smart gas sensor in the mask collected sensing information at low cost and forwarded it to the cloud. The artificial intelligence database in the cloud server used machine learning to realize the rapid processing of data, achieving the effect of real-time monitoring and early warning of epidemics.

It is hoped that through the above-mentioned path, machine learning-assisted gas sensing technology can be upgraded from a single disease detection tool to a part of building a full life cycle health management platform as soon as possible, providing efficient and non-invasive solutions for the early screening of major diseases and management of chronic diseases and promoting the transformation of medical diagnosis models towards community-based and family-based approaches.

## Figures and Tables

**Table 1:** Sensor arrays with machine learning support that correspond to various disorders.

Disease	Sensor Array	Sensor Type	Material	Algorithm	Gas Markers					Parameter		References
Lung cancer and COPD	8 independent sensors form a heterogeneous sensor array	Electrochemical	SnO_2_-based sensitive film, Pt filament carrier + Pd/Al_2_O_3_ catalyst, precious metal electrode (Pt/Au) + liquid electrolyte	KPCA + XGBoost/AdaBoost/RF	Lung cancer	Formaldehyde			0–1000 ppm	Accuracy: 84.75% Sensitivity: 81.36% Specificity: 88.14%		[123]
						Benzene series substances			0–100 ppm			
						Alkanes			1–10,000 ppm			
						CO			1–100 ppm			
						Ethanol			30–5000 ppm			
						Methane			1–100 ppm			
					COPD	Isobutane			300–10,000 ppm			
						Ethanol			30–5000 ppm			
						CO			1–100 ppm			
						Ammonia			30–300 ppm			
	32-channel sensor array	Resistive	Carbon nanotubes, polymer substrates	LDA + SVM	Ethanol				ppb level	AUC = 0.90, 0.91 Sensitivity: 75.0%, 83.3% Specificity: 96.6%, 85.4%		[124]
					Isopropyl alcohol							
					Lipid peroxide-related VOCs							
	12-channel sensor array	Mechanical	Silica/titanium dioxide-based hybrid nanoparticles, commercial polymers	RF classifier	VOCs					Accuracy: 80.9%		[125]
Asthma	Single-channel heterojunction	Resistive	N-type γ-Bi_2_MoO_6_ microspheres	PCA	H_2_S		5 ppb–100 ppm			The response value of 5 ppb H_2_S = 1.5 100 ppb H_2_S is = 4.9		[126]
					NO		>50 ppb					
	Virtual multi-wavelength array	Optical	Quartz, UV-grade fused quartz, metal	PCA + Match/No-match	NO		>40 ppb			Sensitivity 88% AUC-ROC 0.948 Specificity: 89%		[127]
Diabetes	Cross-response model array	Resistive	MOS	Baseline correction + KPCA + AdaBoost + MVRVM + GS/PSO	Acetone					0.1–19.8 ppm		[128]
	A heterogeneous array composed of three independent sensors	Resistive	NiO, CuO, ZnO thin films	KNN, RF, DT, logistic regression, naive Bayes, LDA, ANN, SVM	Acetone		100–2400 ppm			classification accuracy > 99%		[129]
					Ethanol		100–2400 ppm					
	Interdigital electrode structure	Resistive	α-Fe_2_O_3_-MWCNT nanocomposites, Pt interdigital electrodes, Al_2_O_3_, Pt microheaters	CNN, Adam	Acetone		0.5–50 ppm			Accuracy: 85%		[130]
	8-channel sensor array	Resistive	Porous MXene framework	PCA, t-SNE, SVM	C4-C7 aldehydes, ketones, alcohols		5–50 ppm			Accuracy: 91.7% Sensitivity: 88.9% Specificity: 96.8%		[131]
	Scan parameters to obtain multi-dimensional data	Electrochemical	Ni-63 ionization source, parallel metal plate electrode	Two-dimensional wavelet transform + PCA + sparse logistic regression/RF/Gaussian process/SVM	VOCs					<1 year	Specificity: 100% Sensitivity: 92%	[132]
	18 sensor arrays	Resistive	Composite metal oxide							One to four years	Specificity: 82% Sensitivity: 87%	
Hepatic disease	3 sensor modules	Resistive	SnO_2_, WO_3_, Pd	LDA	Alkanes, NO		ppb level			Accuracy: 95–100%		[133]
	Three-electrode system	Electrochemical	Gold nanoparticles, glassy carbon electrodes	PCA, heat map analysis	MBMBP		0.15–0.38 M			——		[134]
	5 commercial MQ sensors + 6 interdigitated sensors	Resistive	Composite metal oxides, WO_3_ nanowire-based	PCA, DFA, SVM	Methanol, dimethyl sulfide, ethanol, toluene					Accuracy: 98.33% AUC = 0.965		[135]
	Five-electrode voltammetry array	Electrochemical	Au, Pd, Pt, glassy carbon (GC), Cu		Cu^2+^ and other electroactive substances					Accuracy: 97.50% AUC = 0.950		
Gastrointestinal disorders	10 thick film MOS sensors	Resistive	Composite metal oxides	RF, neural networks	Aldehydes, acetone, 2-heptanone, p-xylene					AUC = 0.81		[136]
	Single channel ion separation system	Electrochemical	Metal electrode plates	FDA, wavelet transform	VOCs					Accuracy: >75%		[137]
	18 sensor arrays	Resistive	Composite metal oxides	PCA, DFA								
Nervous system disorders	32 chemical sensor array	Resistive	Carbon black-polymer composite materials	PCA, LDA	1-butanol, 2-methylfuran					Sensitivity: 50–70% Accuracy: 68–77%		[138]
	Cross-reactive array consisting of 40 sensors	Resistive	GNPs, SWCNTs	DFA	Benzaldehyde, phenylacetone			ppb to ppm level		Accuracy: 81% Sensitivity: 79% Specificity: 84%		[139]
Nephropathy	23-unit metal–organic frameworks (MOFs) array	Resonant	Metal–organic framework	CLAC calculation, SVD, iterative numerical model	NH_3_			3.32 ± 2.19 ppm		Accuracy: 100%		[140]
	The spiral porous structure printed by DLP 3D printing	Optical	NAGA, Gly, choline chloride, BCG	CNN	NH_3_			0.5–10 ppm		Accuracy: 96.5%		[141]
	6 sensor array	Resistive	Composite metal oxide	PCA, HCA, SVM, PLS	Dichloromethane, 6-nitro-2-picoline, 4-amino-4H-1,2, 4-triazole, styrene, limonene			5–20,000 ppm		Accuracy: 100%		[142]
Bladder cancer	10-sensor array	Resistive	Polyaniline (substrate material) Fluorine-doped tin oxide (electrode)	PCA, SVM, Kmeans	Benzaldehyde, 2-pentanone, butylbenzene, etc.			25–200 ppm		Accuracy: 96.67% Sensitivity: 100% Specificity: 83.33%		[143]
					piperone			7–50 ppm				
	8 types of metal oxide gas sensor arrays	Resistive	Composite metal oxides	PCA, LDA, SVM, RF, KNN	VOCs					Accuracy: PCA + SVM: 97%; LDA + KNN: 97%; LDA + RF: 94%		[144]
Breast cancer	32-sensor array	Resistive	Carbon nanotubes	KNN, SVM, DT, neural network	VOCs					Accuracy: 91% Sensitivity: 86% Specificity: 97%		[145]
Prostate cancer	Various MEMS gas sensor arrays	Resistive	Composite metal oxides	PCA, SVM, KNN, RF, decision tree, naive Bayes	Ethanol			0.3–100 ppm		Accuracy: 100% Sensitivity: 100%		[146]
	Screen-printed electrode array	Electrochemical	Carbon-based electrodes, gold-based electrodes		Formaldehyde			0.1–208 ppm				
	8 types of MXene-TMDC nanocomposite sensor arrays	Resistive	MXene-TMDC nanocomposites, transition metal dichalcogenides	PCA, hierarchical clustering analysis, CNN, LDA, logistic regression, RF, SVM	Glyoxal 4-Heptanone 2-Pentanone			10–100 ppm		Accuracy: 90% Sensitivity: 98% Specificity: 97%		[147]
